# Structure of Diferrocenyl Thioketone: From Molecule to Crystal

**DOI:** 10.3390/molecules24213950

**Published:** 2019-10-31

**Authors:** Piotr Matczak, Grzegorz Mlostoń, Róża Hamera-Fałdyga, Helmar Görls, Wolfgang Weigand

**Affiliations:** 1 Department of Physical Chemistry, Faculty of Chemistry, University of Lodz, Pomorska 163/165, 90236 Lodz, Poland; 2 Department of Organic and Applied Chemistry, Faculty of Chemistry, University of Lodz, Tamka 12, 91403 Lodz, Poland; grzegorz.mloston@chemia.uni.lodz.pl (G.M.); roza.hamera@gmail.com (R.H.-F.); 3 Institute of Inorganic and Analytical Chemistry, Friedrich-Schiller-University Jena, Humboldtstrasse 8, 07743 Jena, Germany; helmar.goerls@uni-jena.de (H.G.); wolfgang.weigand@uni-jena.de (W.W.)

**Keywords:** ferrocene, thioketone, molecular structure, crystal structure, XRD crystallography, quantum-chemical calculation

## Abstract

Ferrocenyl-functionalized thioketones have recently been recognized as useful building blocks for sulfur-containing compounds with potential applications in materials chemistry. This work is devoted to a single representative of such thioketones, namely diferrocenyl thioketone (Fc_2_CS), whose structure has been determined here for the first time. Both X-ray crystallography and a wide variety of quantum-chemical methods were used to explore the structure of Fc_2_CS. In addition to the X-ray structure determination, intermolecular interactions occurring in the crystal structure of Fc_2_CS were examined in detail by quantum-chemical methods. These methods were also an invaluable tool in studying the molecular structure of Fc_2_CS, from the gas phase to solutions and to its crystal. Intramolecular interactions governing the conformational behavior of an isolated Fc_2_CS molecule were deduced from quantum-chemical analyses carried out in orbital space and real space. Our experimental and theoretical results indicate that the main structural features of an isolated Fc_2_CS molecule in its lowest-energy geometry are retained both upon solvation and in the crystal. The tilt of ferrocenyl groups is only slightly affected by crystal packing forces that are dominated by dispersion. Nonetheless, a network of intermolecular interactions, such as H···H, C···H and S···H, was detected in the Fc_2_CS crystal but each of them is fairly weak.

## 1. Introduction

In the last two decades there has been continuous progress in developing effective methods for the preparation and purification of non-enolizable aryl-, hetaryl-, and/or ferrocenyl (Fc)-functionalized thioketones, along with finding their new applications [1,2]. Numerous representatives of this relatively little known class of organosulfur compounds were encountered as unique building blocks for the synthesis of more complex sulfur-containing compounds. For example, aryl hetaryl thioketones were recognized as prone dipolarophiles and dienophiles in [3 + 2]-cycloadditions and hetero-Diels-Alder reactions, respectively, leading to diverse sulfur heterocycles [3,4,5,6,7,8]. Moreover, many of cycloaddition reactions performed with thioketones were shown to occur via non-concerted, step-wise mechanisms and this fact is of great importance for general discussion on the mechanisms of organic reactions [9,10]. In addition to the new applications of thioketones in organic synthesis, their bioactivity [11,12], complexing properties [13], as well as practical use in colorimetric probes for Cd(II) [14] were also reported.

It is well established that the functionalization of organic molecules with Fc-residues is of special importance for modifications of their physico-chemical properties and biological activity; for that reason, ferrocene (FcH) was recently named as an exceptional molecule [15,16]. There are many Fc-functionalized organic molecules that display a significant role in material and medicinal chemistry, e.g., branched polyethylenimine ferrocene and ferroquine [16,17]. Compared to aryl- and/or hetaryl-functionalized thioketones, their analogues bearing Fc-groups have received less attention despite the fact that the remarkable stability of the latter gives a good opportunity for their exploration in the current organic synthesis. In a series of recent publications we demonstrated that Fc-functionalized thioketones are attractive substrates for the synthesis of diverse Fc-containing *S*-heterocycles with variable ring size, e.g., thiiranes [18,19,20], 1,3-dithiolanes [21,22], 1,3-oxathioles [19] and 4*H*-2,3-dihydrothiophenes [8,23]. One of the most spectacular applications is a recently reported protocol of a multi-step preparation of Fc-substituted ethylenes (ferrocifenes), known as the first organometallic anti-cancer agents [20].

The present work is solely focused on a single representative of Fc-functionalized thioketones, namely diferrocenyl thioketone (Fc_2_CS). Prior to the last few years, this thioketone has not received much attention [24,25] and its experimental and theoretical characterization are far away from being complete. From an experimental perspective, the synthesis of Fc_2_CS was described for the first time a long time ago [24]. However, the classical procedure based on the Friedel-Crafts protocol leads to the desired product in variable yields and was not reproducible in our hands. Only a recently developed procedure based on the exploration of a mixed anhydride leads to excellent results and it can be recommended for the preparation of Fc_2_CS on an even larger scale [18]. Several promising laboratory applications of Fc_2_CS were reported and compared with (het)aryl ferrocenyl analogues [18,23]. Some of its properties were measured [25], but its crystal structure has not been reported so far. From a theoretical perspective based on quantum-chemical calculations, Fc_2_CS has not been explored at all. This is not surprising because the theoretical studies of thioketones bearing a single Fc-group or even two (het)aryl groups are very scarce [26,27,28,29,30]. The aforementioned gaps in the experimental and theoretical characterization of Fc_2_CS have motivated us to obtain part of the missing information about this exceptional thioketone. Thus, we have undertaken a combined experimental and theoretical study that is focused specifically on the structure of Fc_2_CS. First, the crystal structure of Fc_2_CS is determined by single-crystal X-ray diffraction (XRD). Next, quantum-chemical calculations are performed for Fc_2_CS in its molecular and crystal forms. The conformational behavior of both isolated and solvated Fc_2_CS molecule is studied in great detail. The computational treatment of Fc_2_CS within cluster and periodic approaches to its crystal structure allows us to gain an insight into the strength and nature of intermolecular interactions affecting the structure of Fc_2_CS. We also consider the methodological aspect of calculations for the molecule and crystal of Fc_2_CS to provide useful hints on choosing an efficient and accurate computational protocol for modeling of Fc-functionalized thioketones. Finally, it should be stressed again that this work has grown out of our ongoing interest in aryl-, hetaryl- and/or Fc-functionalized thioketones [1,2] and it is a continuation of our previous papers devoted to the computational chemistry of such thioketones [26,27,31,32].

## 2. Results and Discussion

### 2.1. X-Ray Crystallography

**Figure 1:**
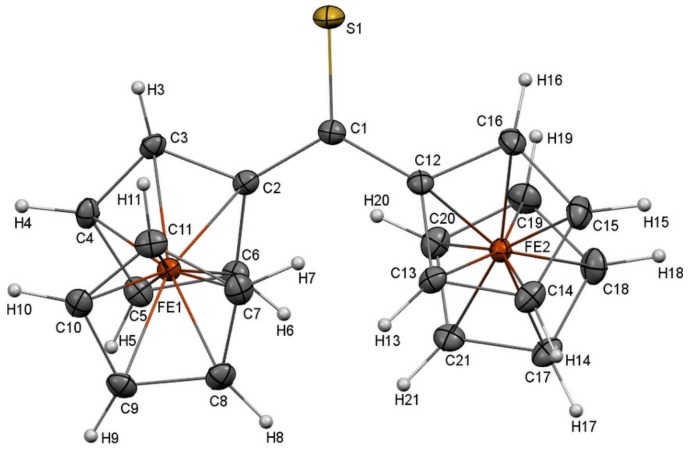
Molecular structure of Fc_2_CS. Displacement ellipsoids are drawn at the 50% probability level.

In the experimental part of this work, the crystal structure of Fc_2_CS was determined by its single-crystal XRD analysis. This analysis revealed that Fc_2_CS crystallizes in the monoclinic space group *P*2_1_/*n*. The asymmetric unit of the unit cell of Fc_2_CS crystal contains one molecule. A summary of the crystal structure of Fc_2_CS is given in Table 1 and a single Fc_2_CS molecule extracted from the crystal structure is shown in Figure 1. The molecular structure of Fc_2_CS exhibits two Fc-groups that are asymmetric with respect to one another. These groups are tilted in the opposite directions, relative to the C2–C1–C12 skeleton. To be precise, the Fc-groups are rotated by 19.4° and 20.1° out of the plane of the C2–C1–C12 skeleton. The cyclopentadienyl (Cp) rings within the Fc-groups are rotated merely by 1.6° and 5.3°, which indicates nearly eclipsed conformations of the Cp-rings.

It is instructive to compare the molecular structure of Fc_2_CS with the structures determined for related crystalline compounds. To this end, a survey of the Cambridge Structural Database (CSD version 5.40, updates up to May 2019 [33]) was conducted. Four crystal structures were found after a search for structures in which a Fc-group was bonded to a thiocarbonyl group and another substituent could be any moiety forming a C–C bond with this C=S group. These crystal structures are identified by the following CSD refcodes and chemical formulas: DUFYAG (C_18_H_18_Fe_1_N_2_O_1_S_2_) [34], JEPVIJ (C_22_H_14_Cr_1_Fe_1_O_5_S_1_) [35], VUTKAX (C_17_H_12_Fe_1_O_5_S_1_W_1_) [36] and VUTKEB (C_29_H_22_Fe_2_O_5_S_1_W_1_) [36]. Their molecular structures are shown in Figure S1 in the Supplementary Materials. The comparison of Fc_2_CS with the Fc-functionalized thioketones present in the four reported structures indicates two important differences between their molecular structures. First, the Cp-rings of Fc_2_CS are less rotated than those of the four related structures. The Cp-rings of JEPVIJ and VUTKEB exhibit a rotation angle of 20.7° and 18.7°, and therefore, they adopt intermediate conformations between staggered and eclipsed. The Cp-rings of DUFYAG and VUTKAX are rotated by 8.1° and 8.9°, respectively. Second, the Cp rings bonded to the C=S group of Fc_2_CS show greater tilt angles outwards the C–C(=S)–C plane than the corresponding Cp-rings of DUFYAG and VUTKAX (their tilt angles are 5.0° and 9.1°, respectively). For JEPVIJ and VUTKEB, the Cp-ring bonded to their C=S group is practically co-planar with this group. A reason for these differences may be that Fc-functionalized thioketones readily act as ligands of transition metals, as it happens in the crystal structures JEPVIJ, VUTKAX and VUTKEB. The presence of other ligands and metal complexation via thiocarbonyl sulfur distort the geometry of these thioketones.

On the other hand, a marked similarity in molecular structure can be observed between Fc_2_CS and its carbonyl analog (Fc_2_CO). The crystal structure of the latter was deposited in the CSD with refcode FEOCKT01 [37]. The Fc-groups of Fc_2_CO are tilted in the opposite directions by 17.1° about the C–C(=O) bonds. The Cp-rings of Fc_2_CO exhibit a nearly eclipsed conformation, with a rotation angle of 5.7°.

### 2.2. Choice of Computational Method

The theoretical part of this work started with establishing a proper computational methodology for studying Fc_2_CS. In order to achieve this, the performance of density functional theory (DFT), represented by the BP-D, BLYP-D, PBE-D, B97-D and TPSS-D functionals, was assessed in predicting the molecular and crystal structure of Fc_2_CS.

First, atomic positions in a single Fc_2_CS molecule extracted from the XRD crystal structure were optimized using the five density functionals in combination with the sequence of three Karlsruhe “def2” basis sets of increasing size (from split valence to triple-ζ valence and to quadruple-ζ valence quality). The same starting geometry of the isolated Fc_2_CS molecule was used in its optimization at all levels of theory. Table 2 presents several geometrical parameters of the optimized molecule. For brevity, only results obtained from the PBE-D functional are shown in this table.

The optimized geometries were compared with the reference molecular geometry taken from the XRD crystal structure. To express the results of comparison quantitatively, the root-mean-square deviation (RMSD) of atomic positions in every optimized geometry relative to the reference geometry was calculated. The RMSD values for the Fc_2_CS molecule optimized using all combinations of five density functionals and three basis sets are listed in Table 3. The collation of RMSD values in each column reveals that the PBE-D functional consistently yields the lowest value. This means that the molecular structure predicted by PBE-D is the closest to the reference molecular geometry of Fc_2_CS. The latter finding is in agreement with previous reports on the robustness of PBE-D in reproducing molecular geometries [38,39,40]. The PBE-D geometry of Fc_2_CS molecule shows very small deviations in angles and slightly less accurate bond lengths (Tables S4–S6). The expansion of basis set from SVP to TZVP is associated with a marked improvement in the reproduction of reference geometry. Further increase of basis set size (that is, from TZVP to QZVP) has a negligible effect on the RMSD values. This proves that the molecular calculations converge fast toward the complete basis set limit. Similar convergence pattern in DFT calculations with TZVP and QZVP was previously detected for geometrical parameters of nitroanilines [41]. As illustrated by the results in Table 2, the experimental bond lengths are reproduced by PBE-D/TZVP with accuracy of 1–2 hundredths of Å. Such accuracy seems to be typical of DFT/TZVP calculations [42].

To situate the aforementioned findings in a wider computational context, an isolated Fc_2_CS molecule was also optimized using a correlated wave function theory (WFT) method. The SCS-MP2 method was selected because it improves the correlation energy of canonical MP2 theory [43,44,45,46,47]. Although SCS-MP2 is one of the simplest and least computationally expensive correlated WFT methods, its computational times are much greater than those of DFT methods (formally *O*(*N*^5^) vs. *O*(*N*^3^)). The RMSD values yielded by SCS-MP2 are appended to Table 3. They show that SCS-MP2 reproduces the reference molecular structure less accurately than the PBE-D method. Even though the SCS-MP2/SVP level luckily affords a RMSD value comparable to that of PBE-D/SVP, the SCS-MP2 method yields much greater RMSD values after the enlargement of basis set. Thus, this method predicts that the isolated Fc_2_CS molecule shows a greater deviation from the molecular geometry observed in the crystal. The main reason for the great deviation of SCS-MP2 geometries is that the SCS-MP2 method systematically underestimates the distance between Fe and Cp-rings (Table S7). This results from the inability of low-order perturbation theory to describe the bonding situation in FcH correctly [48].

Next, the performance of the five density functionals in predicting the crystal structure of Fc_2_CS was assessed. The XRD unit cell of Fc_2_CS was used as a starting point for geometry optimization under periodic boundary conditions. Both the atomic positions and cell parameters of Fc_2_CS were allowed to fully relax during the geometry optimization. The enormous computational cost of the optimization excluded the application of any basis sets larger than SVP. The calculated unit cells were compared with the XRD unit cell. Table 4 presents two criteria that are taken into account for the assessment of the density functionals. The RMSD between the calculated and experimental atomic positions within the unit cell estimates the accuracy of the intrinsic geometry, orientation and alignment of molecules in the optimized crystal structure of Fc_2_CS. The volume of unit cell is a criterion for judging the quality of optimized cell parameters. The RMSD values clearly indicate that the PBE-D functional describes the Fc_2_CS molecules within the unit cell with the greatest accuracy. Again, this is in agreement with previous recommendations on the application of PBE-D to solid-state structures [49,50]. The main reason for the remarkable performance of PBE-D lies in its accurate description of intermolecular distances, leading to the proper orientation and alignment of Fc_2_CS molecules. The RMSD values for individual Fc_2_CS molecules occupying the unit cell prove that BLYP-D, PBE-D and TPSS-D are equally successful in reproducing the intrinsic geometry of Fc_2_CS molecules. Thus, the orientation and alignment of Fc_2_CS molecules within the unit cell are the deciding factors for the superior performance of PBE-D. The alignment of Fc_2_CS molecules is associated with the quality of optimized cell parameters and, in consequence, the volume of unit cell. As shown in Table 4, the PBE-D functional underestimates the volume of Fc_2_CS unit cell merely by about 1%. A reduction by 2.0 to 8.0% occurs for the remaining density functionals. It should be stressed that some underestimation of unit cell volume is actually expected because our calculations neglected zero-point vibrations. It was previously reported that the calculated volume of unit cell for organic crystals is usually reduced by a range up to 2% due to the neglect of vibrational effects [51]. The reduction in the PBE-D volume matches this range perfectly.

The results presented above confirm the credibility of PBE-D for predicting the structure of Fc_2_CS and this functional is highly likely to succeed in reproducing other properties of Fc_2_CS, such as its conformational behavior. There are indeed good grounds for such a supposition because PBE-D was successful in calculating conformational energies of other organic compounds [52,53]. Accordingly, PBE-D is used in the next part of this work. For comparison purposes, the SCS-MP2 method will be adopted in part of molecular calculations where it is computationally feasible.

### 2.3. Gas-Phase Structure

**Figure 2:**
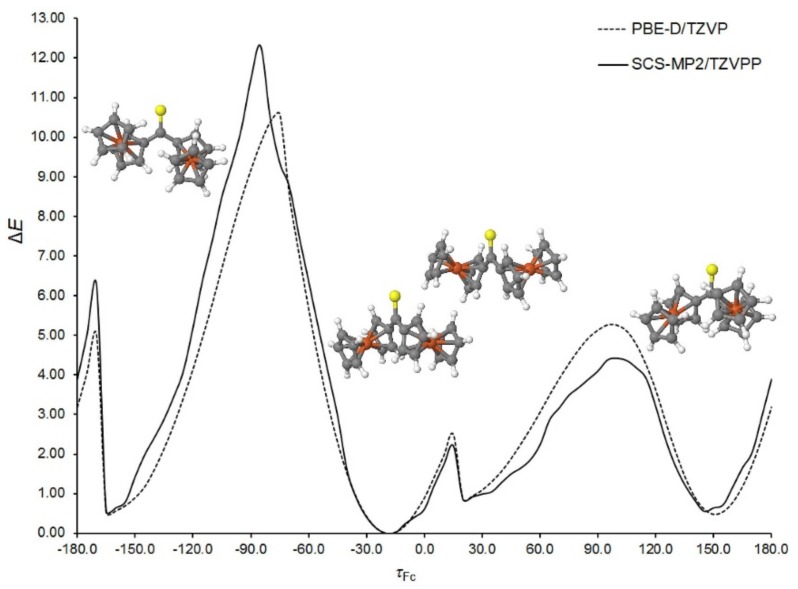
Energy of an isolated Fc_2_CS molecule as a function of the dihedral angle describing the rotation of Fc-group (*τ*_Fc_, in °). The energy (Δ*E*, in kcal mol^−1^) is expressed relative to its lowest value at each level of theory. The schematic illustrations of the PBE-D/TZVP geometries corresponding to the minima of Δ*E* are also presented.

The key aspect of Fc_2_CS molecular geometry is the mutual arrangement of two Fc-groups. They are bonded with the C=S group through single C–C bonds, which opens up an opportunity for their rotation. In consequence, rotational isomerism appears for the Fc_2_CS molecule. To identify possible rotamers, the potential energy surface of an isolated Fc_2_CS molecule was scanned along a coordinate representing the rotation of a single Fc-group about the adjacent C–C bond. The S1–C1–C2–C3 dihedral angle (*τ*_Fc_) was selected to be this coordinate. The Cp-rings of each Fc-group displayed an eclipsed conformation in the starting geometries generated for the scan. The choice of this conformation will be justified further in this subsection. In each point of the scan, all geometrical parameters except *τ*_Fc_ were allowed to fully relax at the PBE-D/TZVP and SCS-MP2/TZVPP levels of theory. The scan is depicted in Figure 2. To facilitate its analysis, the energies of optimized geometries are expressed relative to the lowest-energy geometry for which the relative energy (Δ*E*) amounts to zero. It is easy to notice that there are four energy minima along the *τ*_Fc_ coordinate. Two of them (at around −170° and 150°) are equivalent due to symmetry reasons. All the minima fall into a very narrow range of ca. 1 kcal mol^−1^. The structures corresponding to the minima possess their Cp-ring in nearly eclipsed conformations. Traversing the scan along *τ*_Fc_ is accompanied by the relaxation of another Fc-group; the S1–C1–C12–C16 angle varies between ca. −33° to ca. 29°. From Figure 2 it can also be deduced that the Fc-rotation is associated with the occurrence of several energy barriers. Although the height of each barrier non-systematically varies with the level of theory, both levels agree that the highest barrier appears at *τ*_Fc_ of ca. −90°. Its height amounts to 10.6 and 12.3 kcal mol^−1^ at the PBE-D/TZVP and SCS-MP2/TZVPP levels, respectively. Such a high barrier hinders the fast rotation of Fc-group. The occurrence of this barrier results from the adoption of perpendicular arrangement by the *π* bonds of the C=S group and the adjacent Cp-ring of the revolving Fc-group. In this case another Cp-ring of the revolving Fc-group is oriented downward from the S1 atom. On the other hand, the barrier at around *τ*_Fc_ = 15° is small enough (ca. 2.5 kcal mol^−1^) to allow for an easy interconversion between the neighboring rotamers.

**Figure 3:**
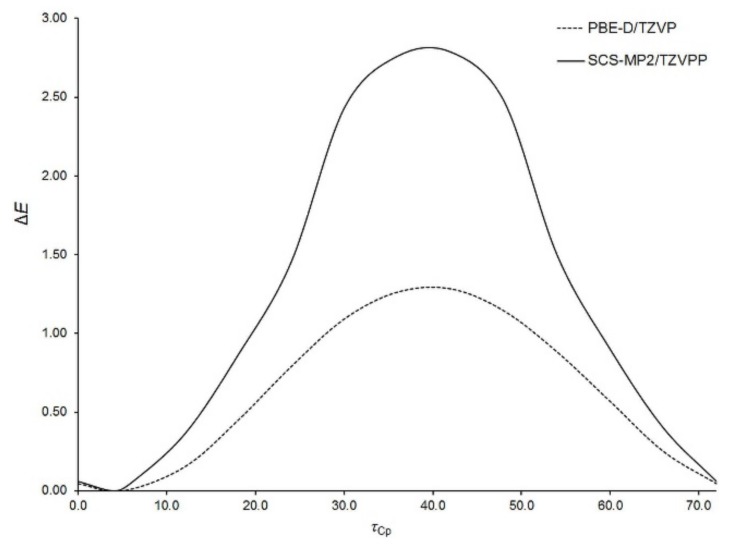
Energy of an isolated Fc_2_CS molecule as a function of the dihedral angle describing the rotation of Cp-rings within one of the Fc-groups (*τ*_Cp_, in °). The energy (Δ*E*, in kcal mol^−1^) is expressed relative to its lowest value at each level of theory.

Aside from the rotation of Fc-group as a whole, the internal rotation of Cp-rings within the Fc-group is also possible. With the aim of characterizing this kind of rotation, the potential energy surface of an isolated Fc_2_CS molecule was scanned along a coordinate describing Cp-ring rotation within one of the Fc-groups. This coordinate was defined as the dihedral angle (*τ*_Cp_) between C3, C11 and the centers of their Cp-rings. The *τ*_Cp_ angle was varied only from 0° to 72° due to the five-fold symmetry of Cp-ring. The resulting relaxed scan calculated at two levels of theory is presented in Figure 3. 

The energy minimum appears at *τ*_Cp_ of ca. 4° and the maximum is observed around 40°. The former corresponds to a nearly eclipsed conformation of Cp-rings and the latter is close to their staggered conformation. Our calculations indicated that the maximum is the transition state for Cp-ring rotation in Fc_2_CS. This is in line with the experimental [54,55] and theoretical [56,57] findings on Cp-ring rotation in an isolated FcH molecule. The eclipsed conformation of Cp-rings in FcH is favored by 0.9 kcal mol^−1^ [55]. The energy barrier of Cp-ring rotation in Fc_2_CS is estimated to be of 1.3 kcal mol^−1^ at the PBE-D/TZVP level. Thus, this barrier is slightly higher than in FcH. The height of the barrier is severely overestimated by the SCS-MP2 method even though the spin scaling lowers this height, compared to that yielded by MP2 [56].

**Figure 4:**
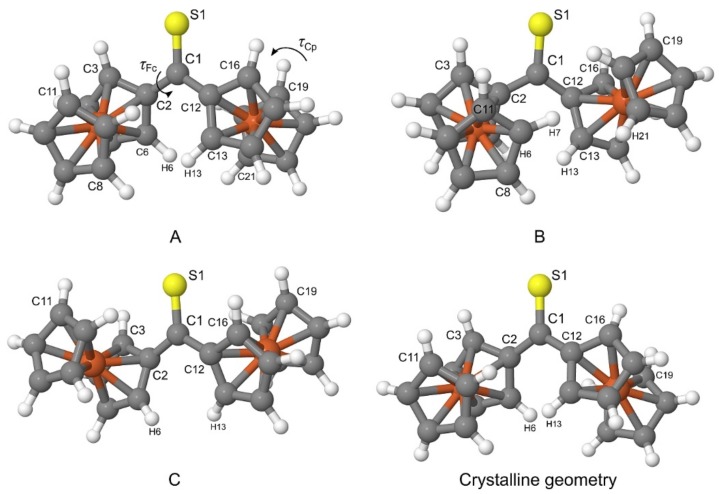
Optimized geometries of three Fc_2_CS rotamers (**A**–**C**) and the XRD molecular geometry of Fc_2_CS. Colors coding individual elements and numbering of selected atoms are the same as in Figure 1 (a version of the present figure with all atoms numbered can be found in the Supplementary Materials, Figure S2).

The PBE-D/TZVP geometries corresponding to three non-equivalent minima in Figure 2 were re-optimized at the PBE-D/QZVP level of theory. For the re-optimized rotamers, their harmonic vibrational frequencies were calculated at the same level of theory to confirm that the rotamers were true minima on the potential energy surface of Fc_2_CS. The application of the QZVP basis set allowed us to practically eliminate the effect of basis-set incompleteness from the PBE-D results. The re-optimized rotamers are schematically depicted in Figure 4 and they will be denoted by letters **A**, **B** and **C**. The calculated geometrical, energetic and electric properties of **A**–**C** are summarized in Table 5. 

As shown in Figure 4, **A** and **C** possess their Fc-groups tilted to the opposite directions, while **B** displays its Fc-groups oriented in the same direction. All three rotamers lie close to each other in energy. **A** is identified as the lowest-energy rotamer (Δ*E* = 0). The tilt of both Fc-groups to the same direction destabilizes **B** merely by 0.5 kcal mol^−1^. The lowest stability is attributed to **C**, which is 0.8 kcal mol^−1^ higher in energy than **A**. The addition of the corrections for the zero-point vibrational energy (ZPVE) to Δ*E* do not change the sequence of rotamers’ stability **A** > **B** > **C**. As evidenced by *τ*_Cp_, the Cp-rings in the three rotamers adopt nearly eclipsed conformations. The stability of the rotamers is related to the magnitude of their dipole moment *μ*. An increase in the stability is accompanied by the growing values of *μ*.

The geometries exhibiting minimal energies in Figure 2 were additionally re-optimized using SCS-MP2/QZVPP. This level of theory proves the occurrence of rotamers **A**–**C** and their selected parameters are appended to Table 5. Both PBE-D/QZVP and SCS-MP2/QZVPP predict the same sequence of rotamers with respect to their stability. Moreover, both levels agree well in their Δ*E* values of rotamers **B** and **C**.

Finally, it is necessary to compare the most stable rotamer in the gas phase with the crystalline geometry of Fc_2_CS. Out of the three rotamers found in the gas phase, **A** indeed bears the strongest resemblance to the crystalline geometry (see Figure 4). The tilt of Fc-groups in the opposite directions changes only slightly between the gas phase and the crystal. Similarly, the Cp-rings of Fc-groups demonstrate nearly eclipsed conformations for both forms of Fc_2_CS. To be precise, the distortions of *τ*_Fc_ and *τ*_Cp_ in **A** from the corresponding values in the crystal do not exceed 2.2°.

Our calculations quite naturally predict that two Fc-groups of **A** are perfect mirror images but such molecular symmetry seems to be violated in the crystal. Nonetheless, the above comparison clearly proves that the transition of Fc_2_CS molecule from the gas phase to the crystal leads to a minor change in its geometry. This also indicates that the crystal packing affects the molecular geometry of Fc_2_CS only slightly.

### 2.4. Intramolecular Interactions

Even though the Δ*E* energies of gas-phase rotamers **A**–**C** fall into a very narrow range (<1 kcal mol^−1^), it is feasible to dissect main factors diversifying the stability of individual rotamers and, consequently, determining the molecular structure of Fc_2_CS. On a chemical basis, such factors may be understood as intramolecular interactions of different kinds. Here, the interactions occurring within the isolated molecule of Fc_2_CS were identified from distinct theoretical perspectives, such as orbital and topological ones, in order to provide an overall picture of factors affecting the molecular structure of Fc_2_CS.

An orbital-based viewpoint on intramolecular interactions was embodied in the analysis of molecular wavefunction in terms of localized orbitals of near-double occupancy. Accordingly, the natural bond order (NBO) analysis of **A**–**C** was performed and its results are summarized in Table 6. First, the most accurate possible Lewis-like description of electron density was obtained for each rotamer, using the NBO method. Such a description assumed a localized assignment of electrons to bonding and non-bonding orbitals. The percentage of the total electron density not covered by the optimal Lewis-like description of each rotamer (%NL) is presented in Table 6. %NL varies marginally from **A** to **B**, whereas the %NL value of **C** is clearly smaller. This suggests that some corrections to the simple Lewis-like description have an important influence on the greater stability of **A** and **B** relative to **C**. Such corrections include electron delocalizations from donor (occupied, Lewis-type) into acceptor (vacant, non-Lewis-type) NBOs. Second-order perturbative treatment of delocalization corrections allowed us to get a close look at the energetics of donor-acceptor delocalizations in **A**–**C**. The donor-acceptor *π*→π* delocalizations between each Fc-group and the C=S group turned out to be crucial for the relative stability of **A**–**C**. Table 6 shows the energy (*E*^(2)^) associated with these delocalizations. The negative sign of *E*^(2)^ indicates that the delocalizations are stabilizing. The *E*^(2)^_Fc→C=S*_ energy is attributed to all delocalizations from the donor π-type NBOs of a given Fc-group to the acceptor π*-type NBO of the C=S group. The *E*^(2)^_C=S→Fc*_ energy is related to all delocalizations from the donor *π*-type NBO of C=S to the acceptor π*-type NBOs of Fc-group. The former always yields greater stabilization. Moreover, the magnitude of *E*^(2)^_Fc→C=S*_ indicates that the stabilizing effect of the Fc→C=S* delocalizations decreases in the sequence **A** > **B** > **C**. This can be deduced from the *E*^(2)^_Fc→C=S*_ values in Table 6 if these are summed up for **B** and doubled for **A** and **C**. The sequence of rotamers ordered according to their total *E*^(2)^_Fc→C=S*_ values is identical to that established in the previous subsection. Thus, the *π*→π* delocalizations between the Fc-groups and the C=S group explain the relative stability of individual rotamers. These delocalizations can be essentially classified as “conjugative” in character, despite the non-coplanarity of the C=S group and the adjacent Cp-rings. The “conjugative” delocalizations are also manifested in the changes of bond lengths. The Fc→C=S* delocalizations lead to an elongation of C=S bond. That is why, the S1–C1 bond (1.666 Å, Table 2) is longer than typical C=S bonds in alkyl thioketones, e.g., dimethyl thioketone shows the C=S bond length of 1.635 Å at the PBE-D/QZVP level. Furthermore, the C–C bonds linking the Fc-groups to the C=S group get compressed due to the “conjugative” delocalizations between these groups. That is why, the C1–C2 bond (1.464 Å, Table 2) is shorter than typical C–C bonds in alkyl thioketones, e.g., dimethyl thioketone possesses the C–C bond lengths of 1.501 Å at the PBE-D/QZVP level.

Rotamers **A**–**C** are differentiated by the tilt of their Fc-groups and these bulky groups are at different distances from one another within **A**–**C**. The Fc-groups of **A** and **B** are in closer proximity than those of **C**. Thus, the former should demonstrate greater steric repulsion. The NBO analysis also offers insights into the steric interactions between the Fc-groups of **A**–**C**. Table 6 shows the steric exchange energy (*E*^(sx)^) that quantifies pairwise steric interactions between filled orbitals. The repulsive character of these interactions is reflected by the positive values of *E*^(sx)^. The *E*^(sx)^_Fc)(Fc_ energy estimates the steric interactions between both Fc-groups of each rotamer. Its values indicate that the steric interactions have a smaller effect on the stability of **A**–**C** than the *π*→π* delocalizations have. As it was expected, **C** is characterized by the smallest value of *E*^(sx)^_Fc)(Fc_. The greatest repulsion between the Fc-groups occurs for **A**. The leading pairwise steric exchange interaction between the Fc-groups involves the *σ*-orbitals of their C–H bonds lying in close proximity, e.g., C6–H6 and C13–H13 for **A** (Figure 4). The *E*^(sx)^_C__–H)(H__–C_ energy shown in Table 6 covers all steric interactions between such C–H bonds for each rotamer.

**Figure 5:**
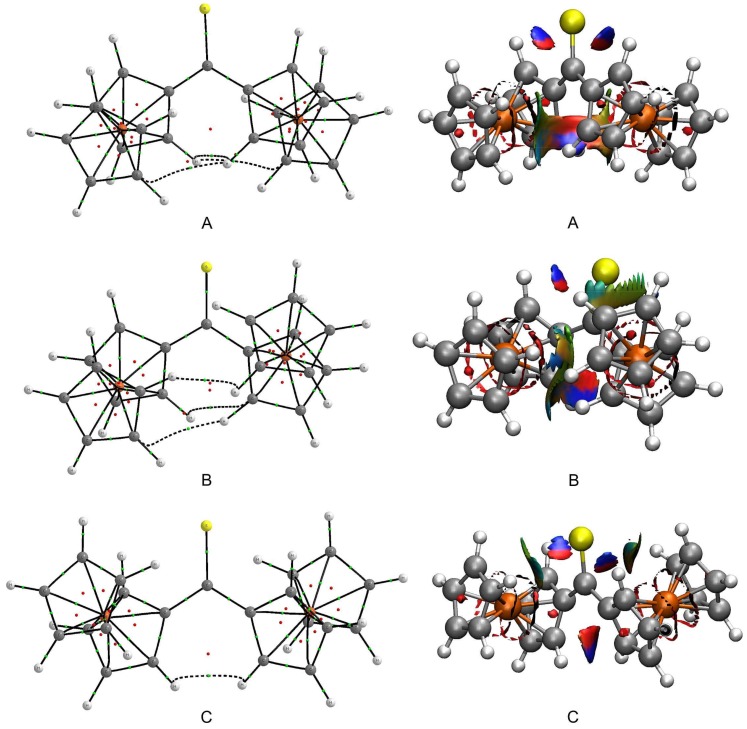
QTAIM molecular graphs and NCI plots for three rotamers (**A**–**C**) of an isolated Fc_2_CS molecule. Bond paths are shown as black lines and their critical points are marked with small green balls. Ring critical points are denoted by small red balls. The bond paths associated with the interactions between the Fc groups are drawn with black dashed lines. The NCI isosurfaces are plotted with a reduced density gradient isovalue of 0.5 a.u. and they are colored from blue to red according to sign(*λ*_2_)*ρ* ranging from negative to positive values, respectively. Colors coding individual elements and numbering of atoms are the same as in Figure 4.

The interactions between the Fc-groups within **A**–**C** were subsequently examined from a perspective based on the topological analysis of electron density in real space. The quantum theory of atoms in molecules (QTAIM) was used to identify subtle interactions between the Fc-groups of each rotamer. QTAIM molecular graphs of **A**–**C** are plotted in Figure 5. This figure proves that the close proximity of Fc-groups within **A** and **B** results in the occurrence of three bond paths linking the Fc-groups (black dashed lines in Figure 5). Two bond paths are assigned to C···H interactions that resemble some unusual kind of hydrogen bonding but they cannot be classified as C–H···C(*π*) [58]. The third bond path corresponds to a H···H interaction. The molecular graph of **C** reveals only a single bond path between two H atoms of Fc-groups. All these interactions were characterized in greater detail using various parameters calculated at the critical point on each aforementioned bond path (Table S8). For these bond paths, their critical point always shows very low values of electron density, its Laplacian and total electron energy density. The Laplacian of electron density and the total electron energy density adopt positive values. Such values of the three parameters describe very weak, closed-shell interactions [59]. Convincing evidence for the closed-shell nature of C···H and H···H interactions within **A**–**C** is additionally provided by other critical-point parameters (see footnotes to Table S8) [60,61].

Our last step towards the detection of even more subtle intramolecular interactions was taken by using the non-covalent interaction (NCI) visualization index. The plots showing NCI isosurfaces for **A**–**C** are presented in Figure 5. The isosurfaces are colored according to the sign of the second Hessian eigenvalue multiplied by the electron density (sign(*λ*_2_)*ρ*). Their blue regions illustrate the occurrence of attractive interactions, while their red regions identify repulsive interactions. The inspection of the NCI isosurfaces for **A**–**C** leads to several findings. First, the blue regions are found between atoms involved in the C···H and H···H interactions, which is in accordance with the QTAIM results. These blue regions border on red regions that in turn are responsible for steric congestion inside the pseudo-rings formed by the C···H and H···H interactions and the surrounding carbon skeleton [31]. Second, the existence of weak stabilizing interactions between the lone electron pairs of S1 and the nearest H atoms of Cp-rings can be observed. The resulting S···H interactions are constrained by the tilt of Fc-groups. Third, there is a vast region, colored in green and yellow, between the Fc-groups of **B**. This region signals extremely weak interactions between the *π*-clouds of the Fc-groups.

### 2.5. Solvated Structure

Having established the structure of an isolated Fc_2_CS molecule, we examine to what extent this structure suffers from the presence of a solvent. The examination of solvent effect is important due to the necessity of carrying out reactions with Fc_2_CS in solution [18]. Three solvents of different polarities, in terms of their dielectric constant and dipole moment, were considered here. In order of increasing polarity, tetrahydrofuran (THF), ethanol (EtOH) and acetonitrile (MeCN) were selected as the solvents. The conductor-like screening model (COSMO) was used to evaluate the effect of these solvents on the Fc_2_CS molecule. In the case of Fc_2_CS solvation by EtOH, we neglected possible hydrogen bonding interactions between the C=S group and one or more hydroxyl groups of EtOH.

**Figure 6:**
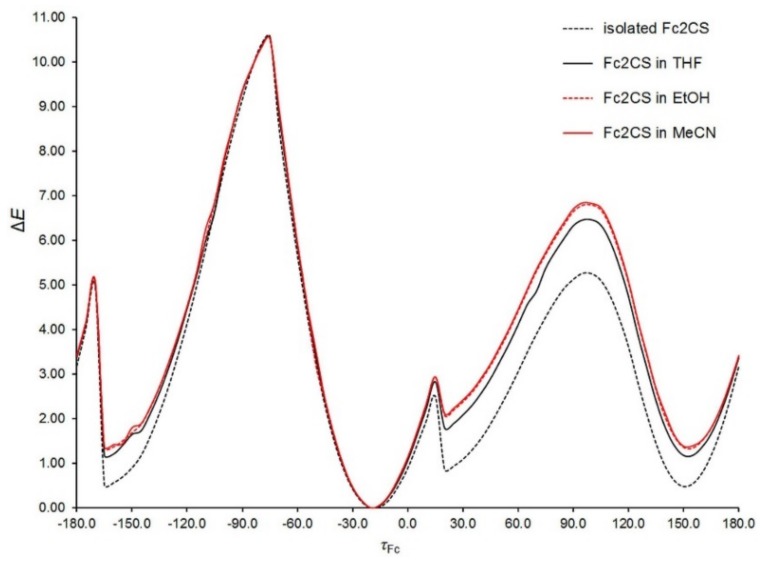
Energy of isolated and solvated Fc_2_CS molecules as a function of the dihedral angle describing the rotation of Fc-group (*τ*_Fc_, in °). The energy (Δ*E*, in kcal mol^−1^) is expressed relative to its lowest value in each medium.

**Figure 7:**
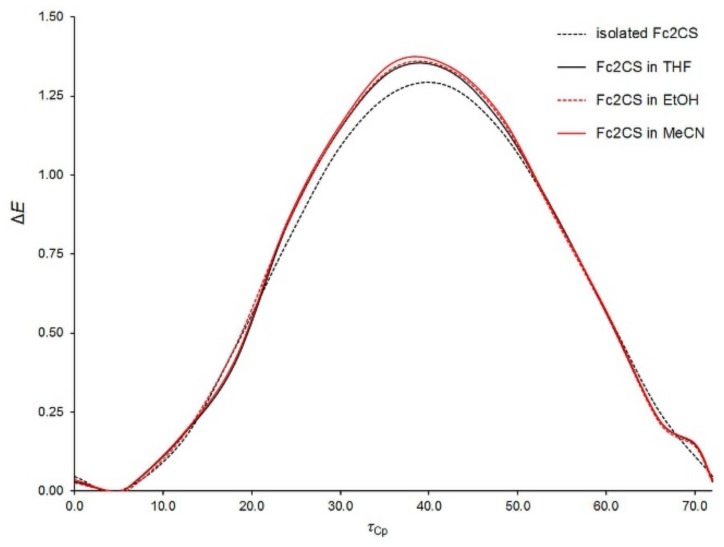
Energy of isolated and solvated Fc_2_CS molecules as a function of the dihedral angle describing the rotation of Cp-ring within the Fc-group (*τ*_Cp_, in °). The energy (Δ*E*, in kcal mol^−1^) is expressed relative to its lowest value in each medium.

The potential energy surface of a Fc_2_CS molecule in the three solvents was scanned along the *τ*_Fc_ and *τ*_Cp_ coordinates at the PBE-D/TZVP level of theory. Except the dihedral angles corresponding to the two coordinates, all other geometrical parameters of solvated Fc_2_CS molecule were allowed to fully relax. The resulting scans are plotted in Figure 6 and Figure 7. In these figures, the PBE-D/TZVP results for the isolated Fc_2_CS molecule are repeated after Figure 2 and Figure 3. It is evident that the conformational behavior of the solvated molecule is fairly similar to that in the gas phase. The solvated molecule demonstrates the minima and maxima of its Δ*E* energy at practically the same *τ*_Fc_ and *τ*_Cp_ angles as the isolated molecule does. By contrast, the solvent effect on the height of energy barriers is noticeable, particularly for the rotation of Fc-group. This effect grows with the increase of solvent polarity. However, the effect of a given solvent on the height of barriers for the Fc-group rotation is not uniform. For instance, the barrier at ca. 95° becomes higher by 14% whereas the barrier at ca. 15° is lowered by half upon solvation by MeCN (Figure 6).

The height of the highest barrier remains practically unchanged if only the Fc-group rotation starts at the *τ*_Fc_ angle corresponding to rotamer **A**. For the rotation of Cp-rings within the Fc-group, its barrier is increased slightly upon solvation (Figure 7). The Cp-ring rotation for Fc_2_CS solvated in MeCN is associated with clearing a barrier of 1.4 kcal mol^−1^, which is merely 5.5% higher than in the gas phase. Figure 7 indicates that the nearly eclipsed conformation of Cp-rings in Fc_2_CS is still favored upon solvation. This is in agreement with the conformation of FcH in solutions. Experimental studies suggested the dominance of eclipsed conformer for FcH in solutions [57,62,63].

For the solvated Fc_2_CS molecule, its geometries representing the minima of Δ*E* in Figure 6 were subsequently re-optimized in three solvents at the PBE-D/QZVP level. The isolated Fc_2_CS molecule could exist as a mixture of three rotamers **A**–**C** and the solvated molecule likewise. Selected parameters of **A**–**C** in three solvents are listed in Table 7. The comparison of *τ*_Fc_ and *τ*_Cp_ in Table 7 to those of the isolated Fc_2_CS molecule (Table 5) reveals that the solvation of Fc_2_CS has a minimal effect on these angles. On closer inspection of *τ*_Fc_ and *τ*_Cp_ in Table 7, it is clear that the angles change marginally with the increase of solvent polarity. 

Bearing in mind that dihedral angles are softer than bond lengths and angles, it can be deduced that the geometrical parameters of a solvated Fc_2_CS molecule are almost insensitive to solvent polarity. The values of Δ*E* + ΔZPVE indicate that **A** is still designated as the preferred rotamer in all three solvents. Furthermore, no variations in the decreasing stability of rotamers **A** > **B** > **C** are observed after adding the solvents. It is worth noting that the preference of **A** is enhanced by the increasing polarity of solvents. There is a clear relation between the dipole moment of **A**–**C** and the solvent polarity. The values of *μ* become higher and higher for all rotamers but the *μ* value of **A** grows to the greatest extent. This is because **A** is the most polar rotamer out of **A**–**C**. In consequence, the strongest stabilization due to electrostatic solute-solvent forces occurs for solvated rotamer **A**.

### 2.6. Crystal Structure

The crystal structure of Fc_2_CS was calculated at the PBE-D/SVP level of theory. Both the cell parameters and the atomic positions of all 168 atoms occupying the unit cell were optimized. The optimization of atomic positions included all atoms because the *P*1 space-group symmetry was exploited in this optimization, due to the limitations of the current version of TURBOMOLE. Calculated geometrical parameters relevant for the crystal structure of Fc_2_CS are summarized in Table 8. The calculated lattice parameters are in close accordance with the values measured using the XRD method. The percentage variations of the calculated lattice parameters from the XRD values do not exceed ±0.5%. These variations result in a slightly too small volume of the computed unit cell (Table 4). The contraction of unit cell is a well-known consequence of the neglect of ZPVE and thermal effects in periodic calculations [64]. In comparison with the XRD structure, four Fc_2_CS molecules occupying the optimized unit cell lie closer to each other. This movement of the Fc_2_CS molecules closer to each other is illustrated by shorter minimal intermolecular distances (min *d*^inter^) of various kind (e.g., H···H, C···H, S···H and C···C). A fundamental deviation of the calculated crystal structure from the XRD one is that the former shows the symmetry-independence of the four Fc_2_CS molecules within the unit cell. This can obviously be explained by the exploitation of *P*1 space-group symmetry in our periodic DFT calculations. Each Fc_2_CS molecule exhibits unique *τ*_Fc_ and *τ*_Cp_ angles and, therefore, the ranges of *τ*_Fc_ and *τ*_Cp_ are given in Table 8. Fortunately, these ranges are quite narrow. The comparison between the calculated and experimental values of *τ*_Fc_ and *τ*_Cp_ reveals that the PBE-D/SVP calculations yielded the crystal structure with somewhat larger asymmetry of Fc-groups in each Fc_2_CS molecule.

**Figure 8:**
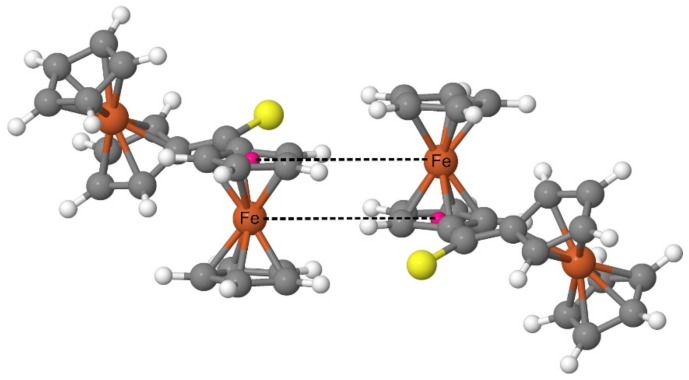
Structural motif extracted from the crystal structure of Fc_2_CS as it was calculated at the PBE-D/SVP level of theory. Geometrical centers of two Cp-rings are marked with small pink balls. Dashed lines link each center with the nearest Fe atom belonging to the neighboring molecule. Colors coding individual elements are the same as in Figure 1.

Both the XRD analysis and the periodic DFT calculations prove that the Fc_2_CS molecules in the crystal structure form a pattern with a highly characteristic arrangement of Fc-groups. Such an arrangement is based on the structure of a dimer comprised of two FcH molecules [65]. The FcH molecules in such a dimer are oriented parallel to their axes running through the geometrical centers of their Cp-rings. Simultaneously, one of the molecules is shifted along the axis running through the geometrical centers of its Cp-rings. As a result of the shift, the Fe atom of each FcH molecule lies on the same plane with one of the Cp-rings of another FcH molecule. The FcH dimer was recognized as a common building block in the crystal structures of ferrocene derivatives [65]. The analysis of the electrostatic potential calculated for the FcH dimer revealed a large stabilizing electrostatic complementarity between the FcH units of this dimer [65]. In the case of Fc_2_CS, the spatial organization of the Fc-groups belonging to neighboring molecules resembles that of the FcH dimer (Figure 8). The calculated crystal structure indeed demonstrates a set of almost right angles formed by the geometrical centers of Cp-rings and their two nearest Fe atoms (one belonging to the same molecule and the other of neighboring molecule). Furthermore, two Fe atoms with their respective Cp-ring centers (linked with dashed lines in Figure 8) lie almost on the same plane.

The calculated crystal structure can also be characterized in terms of energetic quantities. The central quantity to assess the stability of a crystal is its lattice energy. The lattice energy of Fc_2_CS calculated at the PBE-D/SVP level amounts to −40.9 kcal mol^−1^. Its negative value means that the crystal is stable with respect to dissociation into separated gas-phase molecules in their lowest-energy conformation. The effect of crystal packing on the molecular structure of Fc_2_CS can be expressed by the strain energy. For Fc_2_CS, the distortion of its molecular structure in the crystal from that in the gas phase increases the molecular energy by 1.1 kcal mol^−1^ at the PBE-D/SVP level of theory. This is the strain energy that must be compensated by intermolecular interactions in the crystal.

### 2.7. Intermolecular Interactions

**Figure 9:**
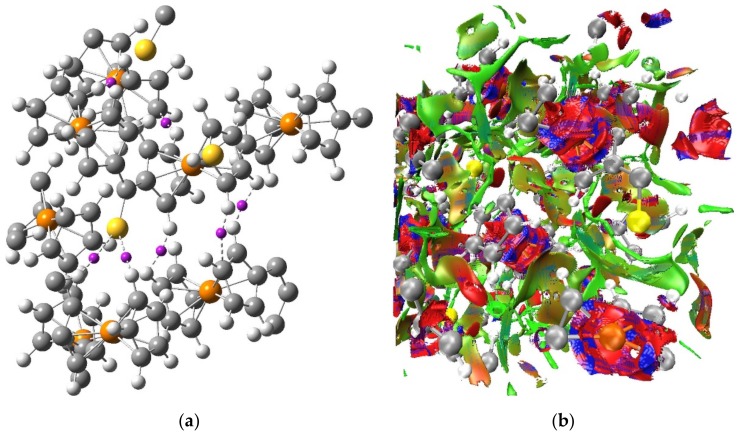
(**a**) QTAIM critical points and (**b**) NCI isosurface for the PBE-D/SVP-optimized unit cell of Fc_2_CS. Only several critical points corresponding to various intermolecular contacts are shown. These critical points are marked with small purple balls. The NCI isosurface is plotted with a reduced density gradient isovalue of 0.5 a.u. and this isosurface is colored from blue to red according to sign(*λ*_2_)*ρ* ranging from its negative to positive values, respectively. Colors coding individual elements are the same as in Figure 1.

For the optimized crystal structure of Fc_2_CS, its electron density calculated at the PBE-D/SVP level of theory was examined using the QTAIM and NCI methods in order to detect intermolecular interactions occurring in the crystal of Fc_2_CS. The QTAIM topological analysis of electron density located plenty of critical points in the intermolecular regions of the crystal structure. After the elimination of all ring and cage critical points, the remaining critical points described three kinds of intermolecular contacts: H···H, C···H and S···H. Figure 9 shows a tiny fraction of the critical points corresponding to the three kinds of intermolecular contacts. Several QTAIM parameters were determined at the critical points of H···H, C···H and S···H contacts to characterize the interactions between the atoms forming these contacts. Irrespective of which contact is considered, the electron density at the critical point is very low in value (<0.01 a.u.) and the Laplacian of the electron density is also small and positive (<0.035 a.u.). The total electron energy density at the critical point is positive but it approaches zero (<0.0015 a.u.). Such values of the parameters testify that the intermolecular H···H, C···H and S···H contacts can be classified as closed-shell interactions and their strength is estimated to be very low. The shortest H···H contact is found at a distance of 2.156 Å (Table 8) and the vast majority of such contacts are no longer than 2.8 Å. Thus, the Fc_2_CS crystal presents slightly longer intermolecular H···H contacts than their typical range 2.18–2.57 Å [66]. This indicates that for Fc_2_CS there are no sufficiently strong crystal packing effects to give rise to short intermolecular H···H contacts and, in consequence, to severe steric overcrowding in the structure of Fc_2_CS. The interatomic distances of S···H contacts in Fc_2_CS lie in the range from 2.821 Å to 3.407 Å. The QTAIM analysis reveals that each S atom in the crystal structure of Fc_2_CS can participate in more than one intermolecular S···H hydrogen bonding interaction. Most of these interactions are detected at longer distances (>3 Å) than typical intermolecular (C=)S···H hydrogen bonding contacts reported for molecular crystals [67]. Formation of several (C=)S···H contacts with the same S atom is known for many crystal structures [67]. For instance, a hydrogen bonding pattern composed of four S···H interactions to a thiocarbonyl group was observed in the crystal structure of thiourea [68]. Based on previous theoretical studies of model complexes [69,70], a single S···H interaction is expected to be rather weak. However, the stabilizing effect of S···H interactions in Fc_2_CS should be strengthened to a certain extent, remembering that a given S atom can be involved in more than one S···H interaction simultaneously. Out of the critical points describing the intermolecular contacts in the crystal structure of Fc_2_CS, those corresponding to C···H interactions are scarcest. The C···H contact of 3.530 Å is in general the longest for which the critical point was detected in Fc_2_CS.

The QTAIM-based picture of a network of weak H···H, C···H and S···H interactions between the molecules of Fc_2_CS crystal is supported by the results of NCI analysis. The NCI isosurface generated for the optimized unit cell of Fc_2_CS is plotted in Figure 9. It is noticeable that the NCI isosurface extends over vast intermolecular regions where the H···H, C···H and S···H contacts appear. This part of the NCI isosurface is colored in green and it signals the occurrence of weak interactions. The lack of any blue domains for the NCI isosurface in the intermolecular regions indicates that no strong interactions are localized between the molecules of Fc_2_CS. The blue and red domains of the NCI isosurface are observed within the Fc-groups. These domains originate from the close proximity of two aromatic rings, which leads to attractive and repulsive interactions between the *π*-clouds of both Cp-rings.

The energetic analysis of the interactions between the molecules of Fc_2_CS crystal was performed to provide a detailed insight into the strength and nature of physical forces lying behind these interactions. The intermolecular interaction energy (*E*^inter^) was determined using a cluster approach to the crystal structure of Fc_2_CS (Figures S3–S5). Within this approach, the structural motif shown in Figure 8 was represented by two Fc_2_CS molecules forming a dimer. Its calculated *E*^inter^ energy amounts to −8.0 kcal mol^−1^ at the PBE-D/SVP level of theory. The negative value of *E*^inter^ means a greater stability of the dimer relative to its molecular constituents. The magnitude of *E*^inter^ suggests that the dimer is rather weakly bound. Nonetheless, this magnitude is sufficient to compensate for the strain energy of both Fc_2_CS molecules (2.2 kcal mol^−1^ in total). The attraction between the molecules in fact far outweighs the increase of their intramolecular energy. The calculations of *E*^inter^ for various dimers extracted from the optimized crystal structure of Fc_2_CS (Table S9) lead to the conclusion that the structural motif from Figure 8 is associated with particularly efficient stabilization.

The localized molecular orbital energy decomposition analysis (LMOEDA) was used to establish the importance of individual physical forces in the interactions between the molecules of Fc_2_CS. LMOEDA results for a dimer representing the structural motif from Figure 8 are summarized in Table 9. The *E*^inter^ energy calculated at the PBE-D/SVP level is decomposed into the electrostatic (*E*^elst^), polarization (*E*^pol^), dispersion (*E*^disp^) and exchange-repulsion (*E*^exch-rep^) components. It is clear that *E*^disp^ is the dominant attractive component, while *E*^pol^ provides only 14.1% of the total attraction between the molecules. The LMOEDA method was subsequently used to decompose the *E*^inter^ energies of other dimers extracted from the optimized crystal structure of Fc_2_CS (Table S9). The *E*^disp^ component turns out to be the dominant attractive component for all dimers. This component represents at least 56.1% of the total attraction between two Fc_2_CS molecules. The dominant role of *E*^disp^ is indeed expected for systems with highly polarizable electron densities, such as the *π*-clouds of Fc-groups in our case. The *E*^pol^ component is always the smallest, providing at most 15.6% of the total attraction. In general, the more strongly two Fc_2_CS molecules interact, the smaller percentage share of *E*^disp^ is observed, and simultaneously, it is accompanied by the greater percentage share of *E*^elst^. Therefore, two Fc_2_CS molecules forming the structural motif from Figure 8 demonstrate the highest percentage share of *E*^elst^ among all dimers studied. This is also in line with the finding on the large stabilizing electrostatic complementarity in the FcH dimer [65], as it was mentioned in the previous subsection.

Finally, the many-body analysis of *E*^inter^ calculated for larger clusters of Fc_2_CS molecules was performed to dissect the energetic effects beyond the interactions within the pairs of Fc_2_CS molecules. A series of clusters composed of three Fc_2_CS molecules was extracted from the optimized crystal structure of Fc_2_CS. The *E*^inter^ energy of each trimer was calculated at the PBE-D/SVP level of theory and then partitioned into two- and three-body contributions (Table S10). Here, the “body” refers to a single Fc_2_CS molecule. Thus, the two-body contribution covers the interactions for all pairs of Fc_2_CS molecules within the trimer. The three-body contribution quantifies the effect of a third molecule on the interaction between the other two molecules within the trimer. Unsurprisingly, the two-body contribution is mainly responsible for the stability of all trimers considered. The three-body contribution accounts for 4.3–17.6% of *E*^inter^. The many-body analysis of *E*^inter^ for a cluster of four Fc_2_CS molecules (that is, a tetramer) produced a negligible four-body contribution. This indicates that the higher-body contributions diminished quickly with the growing number of Fc_2_CS molecules.

## 3. Materials and Methods

### 3.1. Synthesis

The synthesis of Fc_2_CS was performed according to the procedure described in our previous work [18]. The experimental characterization of Fc_2_CS by means of ^1^H nuclear magnetic resonance (NMR) and ^13^C-NMR spectroscopy, infrared spectroscopy, electrospray ionization mass spectrometry and by elemental analysis was also reported there [18].

### 3.2. Crystal Structure Determination

Single-crystal XRD data were collected with a Nonius Kappa-CCD diffractometer (Bruker, Madison, WI, USA) using graphite monochromated Mo-K_α_ radiation (*λ* = 0.71073 Å). Data were corrected for Lorentz and polarization effects; absorption was taken into account on a semi-empirical basis using multiple-scans [71,72,73]. The structure was solved by direct methods (SHELXS-97 [74]) and refined by full-matrix least squares techniques against F_o_^2^ (SHELXL-97 and SHELXL-2014 [75]). All hydrogen atoms were located by difference Fourier synthesis and refined isotropically. All non-hydrogen atoms were refined anisotropically [75]. The Mercury 4.0.0 program [76] was used for XRD structure representation. CCDC-1904100 contains the supplementary crystallographic data for this paper. These data can be obtained free of charge via http://www.ccdc.cam.ac.uk/conts/retrieving.html (or from the CCDC, 12 Union Road, Cambridge CB2 1EZ, UK; Fax: +44 1223 336033; E-mail: deposit@ccdc.cam.ac.uk).

### 3.3. Quantum Chemical Calculations

A series of DFT methods [77] was used to predict the molecular and crystal structure of Fc_2_CS. We considered the generalized gradient approximation (GGA) and meta-GGA classes of density functionals because of their low computational cost. Four GGA functionals (BP [78,79], BLYP [78,80], PBE [81], B97 [82]) and one meta-GGA functional (TPSS [83,84]) were selected and they were combined with the respective Grimme’s dispersion correction [85,86]. The resulting dispersion-corrected functionals (denoted by BP-D, BLYP-D, PBE-D, B97-D and TPSS-D) were combined with the Karlsruhe “def2” basis sets, from the split valence polarization (SVP) to the valence triple- and quadruple-zeta polarization (TZVP and QZVP) basis sets [87,88]. In addition to the five density functionals, the SCS-MP2 method [89] was also used to study the gas-phase structure of Fc_2_CS molecule. Interactions occurring within the rotamers of Fc_2_CS were recognized by means of the NBO analysis [90], the QTAIM analysis [59] and the NCI visualization index [91]. Solvent effects on the molecular structure of Fc_2_CS were established according to the COSMO method [92]. The nature of intermolecular interactions between Fc_2_CS molecules was elucidated by means of the LMOEDA method [93]. The DFT calculations for the crystal structure of Fc_2_CS were carried out using a model with three-dimensional periodicity [94,95,96,97].

The main part of molecular DFT and WFT calculations and all periodic DFT calculations were carried out using the TURBOMOLE 7.2 program [98]. Gaussian 09 D.01 [99] was used to produce input files needed in the molecular NBO, QTAIM and NCI analyses. These analyses were made with the aid of the NBO 6.0 [100], AIMAll 14.06.21 [101] and Multiwfn 3.3 [102] programs, respectively. The electron density obtained from the periodic DFT calculations was analyzed using the Critic2 program [103,104]. Visualizations of computational results were created by AIMAll 14.06.21, VMD 1.9.1 [105] and Jmol 14.2 [106].

Further details and validation of the computational methodology used in this work can be found in Section S1 in the Supplementary Materials. Cartesian coordinates for the optimized molecular and crystal structure of Fc_2_CS are given in Tables S11–S17.

## 4. Conclusions

This work presents a combined experimental and theoretical study on the structure of Fc_2_CS in the form of an isolated molecule, solvated molecule and crystal. In addition to the determination of the molecular and crystal structure of Fc_2_CS, the intra- and intermolecular interactions governing the structure and its stability were characterized. The main findings of the study can be summarized as follows:

1) The isolated molecule features rotational isomerism for Fc-groups and the Cp-rings within these groups. Both DFT and WFT calculations show that the rotation of the Fc-groups produces three rotamers being the local minima of energy. Although the relative energies of these rotamers fall in a very narrow range (<1 kcal mol^−1^), the interconversions between the rotamers are accompanied by several energy barriers, of which the highest far exceeds 10 kcal mol^−1^. Within the Fc-groups, a nearly eclipsed conformation of their Cp-rings is favored by 1.3 kcal mol^−1^. Thus, the barrier of Cp-ring rotation in Fc_2_CS is slightly higher than that in the FcH molecule.
2) The NBO analysis of intramolecular interactions indicates that the stabilization of individual rotamers is mainly governed by the orbital delocalizations between the C=S group and the adjacent Cp-rings. These delocalizations are “conjugative” in character and they provide particularly large stabilization when involve the donor *π*-type orbitals of Fc-groups and the acceptor π*-type orbital of C=S. Compared to the strength of these delocalizations, the steric repulsion between two Fc-groups is a secondary interaction. Surprisingly, this repulsion is highest in magnitude for the preferred rotamer.
3) The DFT calculations involving the COSMO treatment of solvation with THF, EtOH and MeCN lead to three rotamers detected previously in the gas phase. Their geometries are marginally perturbed by the solvents. On the other hand, the solvents produce a noticeable effect on the relative energies of higher-energy rotamers. These rotamers are more and more destabilized by the solvents of growing polarity. This is due to the high dipole moment of the preferred rotamer. Similarly to the isolated rotamers, the solvated ones show nearly eclipsed conformations of their Cp-rings.
4) The XRD structure was reproduced by the periodic DFT optimization with great accuracy. The molecular structure of Fc_2_CS in the crystal corresponds to the lowest-energy rotamer in the gas phase. The deformation of the gas-phase rotamer by crystal packing forces is minor, which is manifested in the small variations of geometrical parameters and in the strain energy amounting to merely 1.1 kcal mol^−1^. The structural motif of the ferrocene dimer is found in the crystal structure of Fc_2_CS.
5) The QTAIM and NCI analyses of the electron density obtained from the periodic DFT calculation predict the formation of a network of H···H, C···H and S···H intermolecular interactions in the crystal structure. If treated individually, these interactions are rather weak, with the dominant role of dispersion, and the associated interatomic distances are often longer than the corresponding lengths of typical H···H, C···H and S···H intermolecular contacts. Nonetheless, their strength is sufficient to compensate for the strain energy of molecules. The S atom of Fc_2_CS is involved in more than one S···H intermolecular contact simultaneously.
6) Our molecular and periodic DFT calculations clearly indicate the robust performance of PBE-D in predicting the structure of Fc_2_CS. In this regard, our study extends the previous recommendations made for PBE-D structures of other classes of chemical systems.

These findings fill in the gaps in the knowledge on the structure of Fc_2_CS and they may also contribute to a better understanding of structural aspects of Fc-functionalized thioketones and other related molecules (e.g. ketones, imines, etc.) in general. In a broader perspective, this study may be important for further development of more complex structures containing the Fc_2_CS building block. In our very recent work we designed a test series of such structures; they were derived from Fc_2_CS and diverse (bisphosphane)Pt(0) complexes [107].

## Figures and Tables

**Table 1:** Crystallographic data and refinement details for Fc_2_CS.

Parameter	Value
Empirical formula	C_21_H_18_Fe_2_S
Formula weight (g mol^−1^)	414.11
Crystal color and habit	Brown prism
Crystal size (mm^3^)	0.112 × 0.108 × 0.102
Temperature (K)	133(2)
Crystal system	Monoclinic
Space group	*P*2_1_/*n*
*a*, *b*, *c* (Å)	10.0041(2), 14.5496(5), 12.2300(4)
*α*, *β*, *γ* (°)	90, 113.340(2), 90
Volume (Å^3^)	1634.48(8)
*Z*	4
Calculated density (g cm^−3^)	1.683
Absorption coefficient (mm^−1^)	1.900
F(000)	848
θ range for data collection (°)	2.24 to 27.45
Index ranges	−12 ≤ *h* ≤ 12, −18 ≤ *k* ≤ 18, −15 ≤ *l* ≤ 15
Reflections collected	11854
Independent reflections [*R*_int_ = 0.0262]	3706
Completeness to θ_max_ (%)	99.7
Max. and min. transmission	0.7456, 0.6280
Restraints and parameters	0, 289
Goodness-of-fit on F^2^	1.061
Final *R* indices [*I* > 2σ(*I*)]	*R*_1_ = 0.0225, w*R*_2_ = 0.0555
*R* indices [all data]	*R*_1_ = 0.0240, w*R*_2_ = 0.0563
Largest difference peak and hole (e Å^−3^)	0.414, −0.308

**Table 2:** Selected geometrical parameters for the optimized isolated Fc_2_CS molecule and for the molecule extracted from the XRD structure of Fc_2_CS crystal. The numbering of atoms corresponds to that shown in Figure 1. Bond lengths are given in Å and angles are in °.

Parameter	PBE-D/SVP	PBE-D/TZVP	PBE-D/QZVP	XRD
S1–C1	1.672	1.666	1.666	1.660(2)
C1–C2	1.470	1.464	1.464	1.461(2)
C2–C3	1.450	1.444	1.444	1.438(3)
Fe1–C2	2.036	2.044	2.044	2.055(2)
Fe1–C7	2.047	2.051	2.050	2.065(1)
C1–C2–C3	124.2	124.3	124.2	124.7(1)
C1–C12–C16	124.2	124.3	124.2	125.5(1)
S1–C1–C2–C3	−18.9	−18.4	−18.4	−19.4(2)
S1–C1–C12–C16	−18.9	−18.4	−18.4	−20.1(2)

**Table 3:** RMSD (in Å) in atomic positions for the optimized isolated Fc_2_CS molecule relative to the reference molecular geometry extracted from the XRD crystal structure of Fc_2_CS.

Method	Basis Set		
	SVP	TZVP ^1^	QZVP ^1^
BP-D	0.294	0.262	0.263
BLYP-D	0.274	0.248	0.245
PBE-D	0.263	0.238	0.239
B97-D	0.310	0.268	0.267
TPSS-D	0.285	0.248	0.250
SCS-MP2	0.268	0.322	0.328

^1^ “PP” sets of polarization functions were used in the SCS-MP2 calculations.

**Table 4:** RMSD (in Å) in atomic positions for the optimized unit cell of Fc_2_CS relative to the XRD unit cell, and the volume of the calculated unit cell (in Å^3^).

Functional	RMSD ^1^	Volume ^2^
BP-D	0.199 (0.121)	1503.71 (92.0)
BLYP-D	0.132 (0.111)	1581.65 (96.8)
PBE-D	0.114 (0.110)	1618.25 (99.0)
B97-D	0.115 (0.115)	1601.94 (98.0)
TPSS-D	0.130 (0.112)	1593.28 (97.5)

^1^ RMSD for a single Fc_2_CS molecule in the optimized unit cell is given in parentheses. ^2^ Percentage of the calculated volume relative to the experimental volume (1634.48 Å^3^) is given in parentheses.

**Table 5:** Selected geometrical (*τ*_Fc_, *τ*_Cp_, in °), energetic (Δ*E*, Δ*E*+ΔZPVE, in kcal mol^−1^) and electric (*μ*, in D) parameters for three rotamers of an isolated Fc_2_CS molecule. ^1^

Parameter	Rotamer		
	A	B	C
*τ*_Fc_^2^	−18.4 (−18.0)	−15.7; −28.6 (−14.5; −33.0)	15.3 (17.7)
*τ*_Cp_^3^	3.8 (3.8)	3.9; 7.2 (4.5; 4.7)	3.8 (4.1)
Δ*E*	0.0 (0.0)	0.5 (0.6)	0.8 (0.9)
Δ*E* + ΔZPVE ^4^	0.0	0.4	0.7
*μ*	3.73 (3.06)	3.50 (2.82)	3.16 (2.64)

^1^ Results without parentheses were obtained at the PBE-D/QZVP level of theory, whereas those in parentheses at the SCS-MP2/QZVPP level. ^2^ Dihedral angles defined by S1–C1–C2–C3 and S1–C1–C12–C16. Both values are listed if different. ^3^ Dihedral angles involving: C3, C11 and two centers of their Cp-rings; C16, C19 and their Cp-ring centers. Both values are listed if different. ^4^ SCS-MP2/QZVPP results are missing due to their prohibitively high computational cost.

**Table 6:** Selected NBO parameters for the rotamers of an isolated Fc_2_CS molecule. All energies are given in kcal mol^−1^.

Parameter	Rotamer		
	A	B	C
%NL ^1^	3.98	3.99	3.82
*E* ^(2)^_Fc→C=S*_^2^	−57.0	−45.2; −52.5	−18.5
*E* ^(2)^_C=S→Fc*_^2^	−4.7	−2.9; −4.7	−11.3
*E* ^(sx)^_Fc)(Fc_^3^	10.8	10.3	7.4
*E* ^(sx)^_C–H)(H–C_^3^	4.2	3.5	2.1

^1^ Percentage of the total electron density not covered by the optimal Lewis-like description of electron density. ^2^ Energy of delocalizations between donor and acceptor orbitals. ^3^ Steric exchange energy between occupied orbitals.

**Table 7:** Selected geometrical (*τ*_Fc_, *τ*_Cp_, in °), energetic (Δ*E* + ΔZPVE, in kcal mol^−1^) and electric (*μ*, in D) parameters for three rotamers of Fc_2_CS solvated in tetrahydrofuran (THF), ethanol (EtOH) and acetonitrile (MeCN).

Solvated Rotamer	Parameter			
	*τ*_Fc_^1^	*τ*_Cp_^2^	Δ*E* + ΔZPVE	*μ*
**A** in THF	−19.0	3.5	0.0	6.11
**B** in THF	−16.7; −26.9	2.8; 7.2	1.1	5.65
**C** in THF	13.6	3.2	1.6	5.02
**A** in EtOH	−19.1	3.4	0.0	6.82
**B** in EtOH	−17.0; −26.4	2.5; 7.2	1.2	6.29
**C** in EtOH	13.1	3.1	1.9	5.56
**A** in MeCN	−19.1	3.4	0.0	6.92
**B** in MeCN	−17.0; −26.3	2.4; 7.2	1.2	6.39
**C** in MeCN	13.0	3.1	1.9	5.64

^1^ Dihedral angles defined by S1–C1–C2–C3 and S1–C1–C12–C16. Both values are listed if different. ^2^ Dihedral angles involving: C3, C11 and two centers of their Cp-rings; C16, C19 and their Cp-ring centers. Both values are listed if different.

**Table 8:** Selected geometrical parameters (*a*, *b*, *c*, min *d*^inter^, in Å; *α*, *β*, *γ*, *τ*_Fc_, *τ*_Cp_, in °) for the Fc_2_CS crystal structure optimized at the PBE-D/SVP level of theory.

Parameter	Value ^1^	Parameter	Value ^1^
*a*	9.964 (−0.4)	min *d*^inter^_H···H_	2.156 (−6.0)
*b*	14.551 (0.0)	min *d*^inter^_C···H_	2.613 (−4.9)
*c*	12.187 (−0.4)	min *d*^inter^_S···H_	2.821 (−4.3)
*α*	90.0 (0.0)	min *d*^inter^_C···C_	3.313 (−0.9)
*β*	113.7 (0.3)	*τ*_Fc_	−19.2 to −19.4; −20.6 to −20.8
*γ*	90.1 (0.1)	*τ*_Cp_	1.8 to 2.1; 6.1 to 6.3

^1^ Percentage variations of calculated geometrical parameters from the corresponding XRD values are given in parentheses.

**Table 9:** Interaction energy and its LMOEDA components for two Fc_2_CS molecules shown in Figure 8. All energies are given in kcal mol^−1^.

Parameter	Value ^1^
*E* ^inter^	−8.0
*E* ^elst^	−7.3 (29.8)
*E* ^pol^	−3.5 (14.1)
*E* ^disp^	−13.8 (56.1)
*E* ^exch-rep^	16.7

^1^ Percentage share of each attractive component with respect to the total attraction is given in parentheses.

## Supplementary Materials

The following are available online. Section S1: Further details and validation of the computational methodology used in the work (Tables S1–S3); Section S2: Additional tables and figures (Tables S4–S17, Figures S1–S5).
