# Supplementary material for: Structure of Diferrocenyl Thioketone: From Molecule to Crystal
Source: Molecules. 2019 Oct 31;24(21):3950. doi: 10.3390/molecules24213950 (PMC6864675; doi:10.3390/molecules24213950)
Supplement: Supplementary file 1 [file molecules-24-03950-s001.pdf]

molecules

Supplementary Materials

Structure of Diferrocenyl Thioketone: From Molecule to Crystal

Piotr Matczak [1,*], Grzegorz Mlostoń [2], Róża Hamera-Fałdyga [2], Helmar Görls [3] and Wolfgang Weigand [3]

[1] Department of Physical Chemistry, Faculty of Chemistry, University of Lodz, Pomorska 163/165, 90236 Lodz, Poland
[2] Department of Organic and Applied Chemistry, Faculty of Chemistry, University of Lodz, Tamka 12, 91403 Lodz, Poland; grzegorz.mloston@chemia.uni.lodz.pl (G.M.); roza.hamera@gmail.com (R.H.–F.)
[3] Institute of Inorganic and Analytical Chemistry, Friedrich-Schiller-University Jena, Humboldtstrasse 8, 07743 Jena, Germany; helmar.goerls@uni-jena.de (H.G.), wolfgang.weigand@uni-jena.de (W.W.)
* Correspondence: piotr.matczak@chemia.uni.lodz.pl; Tel.: +48-426354273

Supplementary Section S1. Further details and validation of the computational methodology used in the work

We decided to use a quantum chemistry method that would be as uniform as possible in both molecular and periodic calculations. It is well known that the computational cost of periodic calculations is generally very high and that is why we restricted the methodology of our periodic calculations to DFT methods, in particular to their GGA and meta-GGA classes. These classes of density functionals do not require the calculation of the exact (that is, Hartree-Fock) exchange and, in consequence, GGA and meta-GGA calculations can be carried out at a lower computational cost than DFT calculations with density functionals belonging to higher rungs of Perdew's Jacob's ladder [S1]. We selected four GGA functionals (BP-D, BLYP-D, PBE-D, B97-D) and one meta-GGA functional (TPSS-D) because they were natively implemented in TURBOMOLE and they afforded us an opportunity for performing relatively fast periodic calculations. The choice of the aforementioned functionals in the periodic calculations brought about the application of these functionals to our molecular calculations.

The BP-D, BLYP-D, PBE-D, B97-D and TPSS-D density functionals contained the respective Grimme's "D3" dispersion corrections with the Becke-Johnson damping function (sometimes denoted as D3(BJ)). The D3(BJ) corrections included both two- and three-body dispersion terms in the periodic calculations, while only two-body term was employed in the molecular calculations. The RI-*J* approximation [S2,S3] was used to obtain a speedup of calculations. This approximation required an auxiliary basis set; the Karlsruhe "def2" auxiliary basis set for RI-*J* [S4] was used in our calculations. The molecular calculations were performed with the *m4* multiple grid for numerical integration, whereas the standard grid of medium quality (*4*) was employed in periodic calculations. The mesh of *k*-points used in the periodic calculations was 5 × 3 × 3 in the directions of lattice vectors *a*, *b* and *c*, respectively. Both the atomic positions and cell parameters (that is, the lengths of lattice vectors and the α, β and γ angles between these vectors) were optimized.

A correlated WFT method was used in part of molecular calculations, with the aim of comparing the performance of PBE-D with that of a WFT representative. The SCS-MP2 method was chosen as the correlated WFT method. The SCS-MP2 correlation energies were calculated using the resolution-of-the-identity (RI) approximation for electron repulsion integrals [S5,S6]. The underlying Hartree-Fock calculations were carried out using the RI approximation for Coulomb and exchange integrals (RI-*JK*) [S7]. Core orbitals were not included in the calculations of SCS-MP2 correlation energies. The SCS-MP2 method was combined with the Karlsruhe "def2" basis sets: SVP, TZVPP and QZVPP. The "PP" sets of polarization functions were recommended for correlated methods combined with the

"def2" basis set of triple-ζ or quadruple-ζ quality [S8]. All SCS-MP2 calculations were done with the aid of TURBOMOLE 7.2.

Solvation effects on the Fc_2CS rotamers were approximated by the COSMO model of solvation. The COSMO model replaces the dielectric medium with a conducting medium. Interlocking spheres are used to generate the cavity. COSMO is a continuum model that is roughly similar to the popular polarizable continuum model (PCM) [S9]. The former uses a simpler, more approximate equation for the electrostatic interaction between the solvent and solute. In fact, COSMO may be considered as a limiting case of the PCM model, where the dielectric constant is set to infinity.

The geometries of isolated and solvated Fc_2CS molecules were optimized with tight convergence thresholds for gradients and displacements ($<10^{-5}$ a.u.). The optimized crystal structure of Fc_2CS fulfilled a looser convergence threshold for gradients ($<10^{-3}$ a.u.).

The values of RMSD between calculated and experimental structures were computed in the VMD 1.9.1 program. The RMSD was defined by

$$\mathrm{RMSD} = \sqrt{\frac{\sum_{i=1}^{N} w_i \left\| p_i^{\mathrm{calc}} - p_i^{\mathrm{exp}} \right\|^2}{N \sum_{i=1}^{N} w_i}} \quad (1)$$

where p_i^{calc} was the position of the ith atom in the calculated structure, p_i^{exp} was the position of the ith atom in the experimental structure, N was equal to 42, w_i was a weighting factor for the ith atom (its mass in our case). The RMSD defined above was minimized using the method of Kabsch [S10]. The resulting minimum values of RMSD are presented in Tables 3 and 4. The RMSD values listed in Tables S4–S6 were calculated using the above formula except p_i was either the ith bond length ($N = 61$) or bond angle ($N = 213$) or torsion angle ($N = 672$) and w_i was always equal to 1.

For the gas−phase rotamers of Fc_2CS, their orbital analysis in terms of NBOs and the topological analyses of their electron density were carried out using their wavefunctions calculated at the PBE-D/QZVP level of theory. These wavefunctions were stored in the respective files generated by Gaussian 09 D.01.

In Subsection 2.3 two levels of theory, that is, PBE-D/TZVP and SCS-MP2/TZVPP, were used to estimate the energy barriers for the rotations of Fc-group and Cp-ring in an isolated Fc_2CS molecule. It is important to verify the ability of the two levels to calculate these barriers with reasonable accuracy. Because there are neither experimental nor high-level theoretical estimates of such barriers for Fc_2CS, it is necessary to consider some other molecules which manifest structural similarities with Fc_2CS and for which accurate rotation barriers are available. In consequence, two molecules are selected. Benzaldehyde is designated as an example of a molecule containing an aromatic ring with the adjacent π-conjugated substituent. Revolving the phenyl ring about the C–C(=O) bond in benzaldehyde roughly resembles the rotation of Fc-group in Fc_2CS. Ferrocene can quite naturally be regarded as a model molecule with Cp-ring rotation. The benzaldehyde molecule is optimized in its two conformations; with either flat or perpendicular arrangement of aldehyde group relative to phenyl ring. The ferrocene molecule is optimized in the eclipsed and staggered conformations of its Cp-rings. The optimizations are performed at the PBE-D/TZVP and SCS-MP2/TZVPP levels of theory. Difference in the energies of two conformations defines the rotation barrier for each molecule. The calculated barriers are listed in Table S1.

Supplementary Table S1. Rotation barriers (in kcal mol^{-1}) calculated for two model molecules at two levels of theory.

Molecule	PBE-D/TZVP	SCS-MP2/TZVPP
Benzaldehyde	9.1	7.5
Ferrocene	0.9	2.1

The reference values of rotation barriers are available for both molecules. For benzaldehyde its rotation barrier is estimated to be 7.7 kcal mol^{-1} [S11]. This is a high-level theoretical value that was

recently proven to be correct [S12]. In the case of ferrocene, the rotation barrier of 0.9 kcal mol^{-1} was deduced from experimental measurements [S13].

The comparison of the calculated barriers (Table S1) with the corresponding reference data reveals that the PBE-D/TZVP level performs excellently for the rotation barrier of ferrocene and it overestimates the rotation barrier of benzaldehyde slightly. For benzaldehyde the barrier calculated at the SCS-MP2/TZVPP level turns out to be in very good agreement with the reference value. Unfortunately, the SCS-MP2/TZVPP level is incapable of reproducing the rotation barrier of ferrocene well. To conclude, the PBE-D/TZVP level affords reliable estimates of rotation barriers for two model molecules and its good performance should be transferred to both kinds of rotation in Fc_2CS. The SCS-MP2/TZVPP level should provide reliable barriers only for the rotation of Fc-group in Fc_2CS.

In Subsection 2.6 the lattice energy was calculated for the crystal of Fc_2CS. The lattice energy ($E^{lattice}$) of Fc_2CS crystal was defined by the following formula:

$$E^{lattice} = (E^{crystal} / Z) - E^{molecule} \quad (2)$$

where $E^{crystal}$ was the energy of the unit cell of Fc_2CS, Z was the number of molecules in the unit cell ($Z = 4$ for Fc_2CS) and $E^{molecule}$ was the energy of an isolated Fc_2CS molecule in its lowest-energy conformation (that is, rotamer **A**). The $E^{crystal}$ and $E^{molecule}$ energies were calculated at the PBE-D/SVP level of theory.

The effect of *k*-point sampling scheme on the calculated value of $E^{lattice}$ is established by performing a series of single-point periodic PBE-D/SVP calculations with various *k*-point sampling schemes. The same crystal structure, previously optimized at the PBE/SVP level, is used in these calculations. The calculated values of $E^{lattice}$ are presented in Table S2. It is evident that the $E^{lattice}$ energy converges very fast and the increase of *k*-point mesh beyond 5×3×3 does not improve the value of $E^{lattice}$.

Supplementary Table S2. The lattice energy ($E^{lattice}$, in kcal mol^{-1}) of Fc_2CS, calculated using various *k*-point sampling schemes.

***k*-point mesh**	$E^{lattice}$
5 × 3 × 3	–40.9
7 × 5 × 5	–40.9
9 × 7 × 7	–40.9
11 × 9 × 9	–40.9

In Subsection 2.7 the intermolecular interactions occurring in the crystal of Fc_2CS were discussed in terms of energetic quantities, such as the interaction energy and its components. The interaction energy (E^{inter}) within pairs, triples or a four of Fc_2CS molecules in the crystal of Fc_2CS was determined at the PBE-D/SVP level of theory. From a computational viewpoint, the molecules constituted a cluster for which the total energy was calculated. Thus, the cluster was formed as either a dimer or a trimer or a tetramer, depending on the number of Fc_2CS molecules (within the cluster approach, individual Fc_2CS molecules can be termed as monomers). The cluster was built using the Fc_2CS unit cell optimized at the PBE-D/SVP level. Part of the atoms occupying the optimized unit cell was translated by lattice vectors to generate whole molecules. The tetramer is shown in Figure S3. Trimers (**T1–T4**, Figure S4) and dimers (**D1–D6**, Figure S5) were obtained by removing certain molecules from the tetramer.

The energies of individual molecules constituting a given *n*-mer (*n* = di, tri, tetra) were subtracted from the energy of the entire *n*-mer in order to obtain E^{inter}. Both the *n*-mer and the individual molecules exhibited their geometries taken from the optimized unit cell of Fc_2CS crystal. In order to remove the basis-set superposition error from the values of E^{inter}, the counterpoise correction proposed by Boys and Bernardi [S14] was employed.

An important issue in calculating interaction energies between molecules is the quality of the basis set applied. SVP is a basis set of modest size and it does not include diffuse functions. To assess the reliability of the SVP basis set in predicting E^{inter} between Fc_2CS molecules, E^{inter} for one selected dimer (denoted as **D1** in Figure S5) was calculated using PBE-D combined with three orbital basis

sets of increasing size: SVP, TZVP and aug-cc-pVDZ [S15]. Only the aug-cc-pVDZ basis set covered a set of diffuse functions. The calculations involved the following numbers of primitive Gaussians: 1624, 2714 and 4032, respectively. The calculated values of E^{inter} are listed in Table S3. The magnitude of E^{inter} at the PBE-D/SVP level is reduced by ca. 5%, compared to the E^{inter} value obtained from PBE-D/aug-cc-pVDZ. The reduction of E^{inter} at PBE-D/SVP level turns out to be surprisingly small despite the moderate size of SVP and the lack of diffuse functions. It should be stressed that the size of the aug-cc-pVDZ basis set is twice and half as large as that of SVP. Thus, the SVP basis set provides a reasonable compromise between accuracy and computational cost. Based on the successful validation of PBE-D/SVP for E^{inter} in **D1**, it can be assumed that this level of theory is able to predict E^{inter} in other *n*-mers reliably.

Supplementary Table S3. Interaction energy and its LMOEDA components between two Fc_2CS molecules of dimer **D1**. The dimer is shown in Figure S5. All energies are given in kcal mol^{-1}.

Basis set	E^{inter}	E^{elst} [1]	E^{pol} [1]	E^{disp} [1]	$E^{exch\text{-}rep}$
SVP	–8.0	–7.3 (29.7)	–3.5 (14.1)	–13.8 (56.1)	16.7
TZVP	–8.1	–7.4 (29.1)	–3.7 (14.8)	–14.2 (56.1)	17.2
aug-cc-pVDZ	–8.3	–7.4 (28.9)	–3.8 (14.9)	–14.3 (56.2)	17.2

[1] Percentage share of each attractive component with respect to the total attraction is given in parentheses.

The calculated E^{inter} energy was also analyzed in more detail using two partitioning schemes. First, the E^{inter} energy of each dimer was partitioned into several terms according to the LMOEDA method. Electrostatic terms, such as nuclear-nuclear, 1-electron and 2-electrons electrostatic interactions, were grouped into the electrostatic component (E^{elst}). The polarization component (E^{pol}) included orbital relaxation effects. The dispersion component (E^{disp}) was composed of the correlation term and the dispersion term calculated using Grimme's empirical dispersion correction included in the PBE-D density functional. Finally, exchange and repulsion terms were joined together into the exchange-repulsion component ($E^{exch\text{-}rep}$).

$$E^{inter} = E^{elst} + E^{pol} + E^{disp} + E^{exch\text{-}rep} \quad (3)$$

The four components calculated using PBE-D and different basis sets are appended to Table S3. It is clear that the percentage shares of E^{elst}, E^{pol} and E^{disp} are practically insensitive to the basis set used.

Second, the many-body analysis was performed for the E^{inter} energy of the tetramer shown in Figure S3 to estimate the strength of individual interactions between the Fc_2CS molecules occupying the unit cell of Fc_2CS crystal. According to this analysis, E^{inter} of a cluster possessing four molecular fragments (*i, j, k, l*) can be partitioned into its two-, three- and four-body contributions (E^{inter}_{n-body}, n = 2, 3, 4).

$$E^{inter} = E^{inter}_{2-body} + E^{inter}_{3-body} + E^{inter}_{4-body} \quad (4)$$

$$E^{inter} = E_{tot}(i,j,k,l) - E_{tot}(i) - E_{tot}(j) - E_{tot}(k) - E_{tot}(l) \quad (5)$$

$$E^{inter}_{2-body} = \sum_{i=1<j}^{4} \left(E_{tot}(i,j) - E_{tot}(i) - E_{tot}(j) \right) \quad (6)$$

$$E^{inter}_{3-body} = \sum_{i=1<j<k}^{4} \left(E_{tot}(i,j,k) - E_{tot}(i,j) - E_{tot}(j,k) - E_{tot}(i,k) + E_{tot}(i) + E_{tot}(j) + E_{tot}(k) \right) \quad (7)$$

$$E^{inter}_{4-body} = E^{inter} - E^{inter}_{2-body} - E^{inter}_{3-body} \quad (8)$$

where *i, j, k, l* indicate individual monomers in the tetramer; $E_{tot}(i)$, $E_{tot}(i,j)$, $E_{tot}(i,j,k)$ and $E_{tot}(i,j,k,l)$ denote the total energies of given monomer, dimer, trimer and tetramer, respectively. The two-body

contribution to E^{inter} expresses the sum of the interaction energies for all pairs of Fc_2CS molecules constituting the tetramer. The three-body contribution to E^{inter} covers the three-body effects on the interaction energies of all trimers formed within the tetramer.

Supplementary Section S2. Additional tables and figures

Supplementary Table S4. RMSD (in Å) in bond lengths for the optimized geometry of an isolated Fc_2CS molecule relative to the corresponding bond lengths of the reference molecular geometry extracted from the XRD crystal structure of Fc_2CS.

Method	Basis Set		
	SVP	TZVP [1]	QZVP [1]
BP-D	0.0826	0.0763	0.0756
BLYP-D	0.0821	0.0759	0.0750
PBE-D	0.0829	0.0767	0.0762
B97-D	0.0798	0.0739	0.0733
TPSS-D	0.0802	0.0743	0.0737
SCS-MP2	0.0960	0.0942	0.0960

[1] "PP" sets of polarization functions were used in the SCS-MP2 calculations.

Supplementary Table S5. RMSD (in °) in bond angles for the optimized geometry of an isolated Fc_2CS molecule relative to the corresponding bond angles of the reference molecular geometry extracted from the XRD crystal structure of Fc_2CS.

Method	Basis Set		
	SVP	TZVP [1]	QZVP [1]
BP-D	1.66	1.16	1.16
BLYP-D	1.57	1.08	1.08
PBE-D	1.51	1.06	1.05
B97-D	1.73	1.11	1.11
TPSS-D	1.62	1.14	1.13
SCS-MP2	2.72	2.71	2.76

[1] "PP" sets of polarization functions were used in the SCS-MP2 calculations.

Supplementary Table S6. RMSD (in °) in torsion angles for the optimized geometry of an isolated Fc_2CS molecule relative to the corresponding torsion angles of the reference molecular geometry extracted from the XRD crystal structure of Fc_2CS.

Method	Basis Set		
	SVP	TZVP [1]	QZVP [1]
BP-D	3.23	2.69	2.67
BLYP-D	3.22	2.65	2.63
PBE-D	2.83	2.35	2.34
B97-D	3.48	2.70	2.68
TPSS-D	3.06	2.35	2.49
SCS-MP2	2.92	3.00	2.99

[1] "PP" sets of polarization functions were used in the SCS-MP2 calculations.

Supplementary Table S7. Selected geometrical parameters extracted from the optimized structure of an isolated Fc_2CS molecule and from the XRD structure of Fc_2CS crystal. The numbering of atoms corresponds to that shown in Figure S2. Bond lengths are given in Å and angles are in °.

Parameter	SCS-MP2/SVP	SCS-MP2/TZVPP	SCS-MP2/QZVPP	XRD
S1–C1	1.647	1.643	1.642	1.660(2)

C1–C2	1.477	1.473	1.470	1.461(2)
C2–C3	1.449	1.445	1.442	1.438(3)
Fe1–C2	1.947	1.939	1.933	2.055(2)
Fe1–C7	1.960	1.950	1.945	2.065(1)
C1–C2–C3	124.3	123.8	123.8	124.7(1)
C1–C12–C16	124.3	123.8	123.8	125.5(1)
S1–C1–C2–C3	–18.4	–18.0	–18.0	–19.4(2)
S1–C1–C12–C16	–18.4	–18.0	–18.0	–20.1(2)

Supplementary Table S8. Distance (*d*, in Å) between atoms linked by a bond path and selected QTAIM parameters (ρ, $\nabla^2\rho$, *H*, DI, in a.u.) at the critical point on the bond path for rotamers **A**–**C** in the gas phase. The numbering of atoms linked by a bond path is explained in Figure S2.

Rotamer/Bond Path	*d*	ρ [1]	$\nabla^2\rho$ [2]	*H* [3]	–*V*/*G* [3]	–λ1/λ3 [4]	DI [5]
**A**/H6···H13	2.078	0.0125	0.0450	0.0019	0.800	0.157	0.019
**A**/C8···H13	2.886	0.0059	0.0196	0.0011	0.724	0.161	0.015
**A**/C21···H6	2.886	0.0059	0.0196	0.0011	0.724	0.161	0.015
**B**/H7···H21	2.286	0.0069	0.0240	0.0012	0.739	0.179	0.014
**B**/C13···H6	2.627	0.0103	0.0384	0.0019	0.757	0.124	0.024
**B**/C8···H13	2.819	0.0063	0.0210	0.0011	0.736	0.168	0.016
**C**/H6···H13	2.196	0.0117	0.0433	0.0020	0.770	0.122	0.016

[1] Molecular electron density. [2] Laplacian of ρ. [3] Total electron energy density $H = V + G$, where *V* is the electron potential energy density and *G* denotes the electron kinetic energy density. [4] Ratio of the lowest λ_1 and highest λ_3 eigenvalues of the Hessian matrix of ρ. [5] Delocalization index between two atoms linked by a bond path.

Supplementary Table S9. Selected distances between atoms belonging to different molecules (d^{inter}, in Å), the interaction energy between the molecules and its LMOEDA components (E^{inter}, E^{elst}, E^{pol}, E^{disp}, $E^{exch\text{-}rep}$, in kcal mol^{-1}) for dimers **D1**–**D6** extracted from the optimized unit cell of Fc_2CS. The dimers are shown in Figure S5.

Dimer	$d^{inter}_{C1\cdots C1}$ [1]	min $d^{inter}_{H\cdots H}$	E^{inter}	E^{elst} [2]	E^{pol} [2]	E^{disp} [2]	$E^{exch\text{-}rep}$
**D1**	8.018	2.156	–8.0	–7.3 (29.8)	–3.5 (14.1)	–13.8 (56.1)	16.7
**D2**	7.388	2.774	–7.6	–4.3 (26.5)	–2.6 (15.6)	–9.5 (57.9)	8.7
**D3**	9.529	2.520	–5.8	–4.5 (24.8)	–2.3 (12.8)	–11.3 (62.4)	12.3
**D4**	11.822	2.127	–3.6	–3.5 (22.7)	–2.1 (13.9)	–9.7 (63.4)	11.8
**D5**	11.553	4.929	–0.4	–0.1 (27.0)	0.0	–0.3 (73.0)	0.0
**D6**	15.946	7.487	–0.02	0.07	–0.01	–0.08	0.00

[1] Numbering of atoms is explained in Figure S2. [2] Percentage share of each attractive component with respect to the total attraction is given in parentheses.

Supplementary Table S10. Distances between two C1 atoms belonging to different molecules ($d^{inter}_{C1\cdots C1}$, in Å), the interaction energy between the molecules and its two- and three-body contributions (E^{inter}, $E^{inter}_{2\text{–body}}$, $E^{inter}_{3\text{–body}}$, in kcal mol^{-1}) for trimers **T1**–**T4** extracted from the optimized unit cell of Fc_2CS. The trimers are shown in Figure S4.

Trimer	$d^{inter}_{C1\cdots C1}$ [1]	E^{inter}	$E^{inter}_{2\text{–body}}$	$E^{inter}_{3\text{–body}}$

**T1**	7.388, 8.018, 11.822	−25.4	−24.3	−1.1
**T2**	8.018, 9.529, 11.553	−18.9	−17.8	−1.1
**T3**	7.388, 9.529, 15.946	−16.9	−15.0	−1.9
**T4**	11.553, 11.822, 15.946	−4.9	−4.0	−0.9

[1] Numbering of atoms is explained in Figure S2.

Supplementary Table S11. Cartesian coordinates (x, y, z, in Å) for isolated rotamer **A** optimized at the PBE-D/QZVP level of theory.[1]

**Atom**	**x**	**y**	**z**
Fe	4.124384	5.130033	4.879875
Fe	7.673692	5.717160	1.053458
S	5.753265	8.772079	3.347372
C	5.825120	7.118155	3.158819
C	4.603603	6.321428	3.289550
C	3.397270	6.783497	3.934519
H	3.285356	7.766614	4.377807
C	2.430438	5.737457	3.899109
H	1.434500	5.776359	4.329454
C	3.015984	4.611257	3.237060
H	2.539432	3.650651	3.064977
C	4.351423	4.956227	2.868193
H	5.044253	4.322786	2.328198
C	5.716416	5.161017	6.170580
H	6.653038	5.673179	5.970885
C	5.398916	3.814847	5.800899
H	6.064563	3.113132	5.307581
C	4.042431	3.558147	6.185285
H	3.497199	2.633254	6.024796
C	3.523845	4.747623	6.793123
H	2.514729	4.883159	7.169834
C	4.558935	5.739330	6.781978
H	4.473214	6.759198	7.143233
C	7.109232	6.478952	2.864599
C	7.482608	5.081259	2.973793
H	6.852335	4.286166	3.352369
C	8.839004	4.943715	2.550224
H	9.398130	4.013630	2.508467
C	9.318411	6.235179	2.160784
H	10.302886	6.454616	1.759250
C	8.264520	7.175347	2.349940
H	8.286719	8.238335	2.137530
C	7.878844	4.487837	−0.567669
H	8.502684	3.600415	−0.611592
C	8.288179	5.822572	−0.890323
H	9.278773	6.124426	−1.216087
C	7.171804	6.694487	−0.670355
H	7.166406	7.772636	−0.794932
C	6.073609	5.897714	−0.215311
H	5.097912	6.270106	0.082495
C	6.508710	4.535396	−0.150530
H	5.909904	3.687051	0.166638

[1] Total energy amounts to −3735.66147222102 hartree.

Supplementary Table S12. Cartesian coordinates (x, y, z, in Å) for isolated rotamer **B** optimized at the PBE-D/QZVP level of theory.[1]

Atom	x	y	z
S	0.079612	0.513675	−0.792312
C	0.344926	0.067048	0.789684
Fe	−2.125318	−1.818969	1.374209
C	−2.769166	−2.209087	−0.520293
C	−1.523087	−2.867676	−0.272444
C	−1.699302	−3.753886	0.838316
C	−3.056417	−3.641063	1.281880
C	−3.718514	−2.688654	0.440655
C	−2.116957	0.196982	1.528673
C	−2.919322	−0.363724	2.567078
C	−2.099243	−1.249680	3.338156
C	−0.779924	−1.223437	2.788300
C	−0.769400	−0.309449	1.667401
H	−2.940305	−1.446693	−1.273215
H	−0.596237	−2.668365	−0.802737
H	−0.938473	−4.387436	1.284290
H	−3.498564	−4.165705	2.123255
H	−4.749952	−2.363418	0.536076
H	−2.425667	0.893785	0.756619
H	−3.977827	−0.177983	2.721685
H	−2.428979	−1.858668	4.174677
H	0.066186	−1.810337	3.126428
Fe	2.974824	−1.400199	1.764025
C	2.486413	−3.175417	2.663967
C	2.038861	−3.163245	1.303889
C	3.176989	−2.962159	0.460674
C	4.332832	−2.851889	1.301115
C	3.906100	−2.982549	2.662811
C	2.060980	0.119020	2.762887
C	3.477886	0.262556	2.849137
C	4.000135	0.354824	1.518497
C	2.908936	0.275224	0.605402
C	1.686719	0.143506	1.361165
H	1.862360	−3.299481	3.543977
H	1.007025	−3.226583	0.975911
H	3.161336	−2.876674	−0.621179
H	5.349620	−2.670932	0.966685
H	4.543189	−2.922849	3.539733
H	1.375904	0.040539	3.599331
H	4.059586	0.271906	3.766106
H	5.049259	0.436337	1.251266
H	2.953976	0.302378	−0.477657

[1] Total energy amounts to −3735.66074055444 hartree.

Supplementary Table S13. Cartesian coordinates (x, y, z, in Å) for isolated rotamer **C** optimized at the PBE-D/QZVP level of theory.[1]

Atom	x	y	z
Fe	3.655880	5.923983	5.039087
Fe	8.052683	6.573641	1.072501

S	5.773126	8.707754	3.369752
C	5.832739	7.051528	3.161128
C	4.622806	6.234498	3.266322
C	3.273372	6.752644	3.228188
H	3.029557	7.794930	3.053080
C	2.368565	5.676022	3.465160
H	1.286495	5.754005	3.507549
C	3.133503	4.484632	3.683012
H	2.733502	3.504595	3.925533
C	4.517750	4.819544	3.572237
H	5.346702	4.141597	3.738348
C	4.782990	6.826583	6.486849
H	5.728395	7.319244	6.278263
C	4.600097	5.440646	6.792814
H	5.388184	4.701678	6.898981
C	3.192280	5.193738	6.893130
H	2.724060	4.234265	7.090391
C	2.508163	6.429827	6.649230
H	1.431610	6.569048	6.631303
C	3.494015	7.440164	6.396729
H	3.304392	8.476672	6.137400
C	7.096070	6.373782	2.866358
C	7.296808	5.086593	2.225984
H	6.515962	4.410260	1.898687
C	8.699393	4.890942	2.039070
H	9.164262	4.030889	1.566331
C	9.380860	6.046851	2.540572
H	10.453730	6.211948	2.519556
C	8.405611	6.964462	3.031149
H	8.577216	7.948354	3.454145
C	8.533558	6.348851	−0.903300
H	9.063735	5.501714	−1.327593
C	9.134368	7.534877	−0.366810
H	10.198806	7.742401	−0.315917
C	8.085280	8.380988	0.123126
H	8.206924	9.335124	0.625559
C	6.840704	7.716748	−0.112971
H	5.867130	8.075171	0.208983
C	7.113908	6.462732	−0.746039
H	6.377300	5.716955	−1.028338

[1] Total energy amounts to −3735.66024537148 hartree.

Supplementary Table S14. Cartesian coordinates (x, y, z, in Å) for isolated rotamer **A** optimized at the SCS-MP2/QZVPP level of theory.[1]

Atom	x	y	z
Fe	4.130000	5.137301	4.812368
Fe	7.668790	5.708213	1.120580
S	5.756853	8.677272	3.332926
C	5.828231	7.047059	3.149406
C	4.606648	6.241511	3.298638
C	3.411984	6.711928	3.955838
H	3.287127	7.694953	4.374462

C	2.450068	5.653717	3.956067
H	1.473485	5.691435	4.409382
C	3.031241	4.521638	3.319760
H	2.569297	3.556016	3.195726
C	4.364967	4.865037	2.919084
H	5.043316	4.223294	2.389194
C	5.707664	5.274817	5.941492
H	6.625180	5.789274	5.707117
C	5.403251	3.909547	5.632372
H	6.057310	3.206758	5.144846
C	4.064862	3.645736	6.059712
H	3.532202	2.717553	5.938808
C	3.541682	4.846545	6.640660
H	2.545731	4.978942	7.028457
C	4.557276	5.851333	6.569228
H	4.460536	6.875340	6.888240
C	7.113262	6.403208	2.837028
C	7.476370	5.002900	2.903654
H	6.861006	4.203441	3.271024
C	8.830826	4.873382	2.449830
H	9.375480	3.947846	2.360761
C	9.306078	6.164311	2.086666
H	0.271371	6.385839	1.662675
C	8.256540	7.109068	2.312517
H	8.292144	8.167542	2.123994
C	7.850385	4.542409	−0.426286
H	8.462267	3.660052	−0.509396
C	8.262424	5.882819	−0.720720
H	9.239472	6.183460	−1.059581
C	7.164661	6.755190	−0.437836
H	7.169635	7.828913	−0.520373
C	6.074375	5.956593	0.034744
H	5.118195	6.325024	0.368530
C	6.498386	4.588239	0.035775
H	5.912097	3.740977	0.348508

[1] Total energy amounts to −3733.2173681494 hartree.

Supplementary Table S15. Cartesian coordinates (x, y, z, in Å) for isolated rotamer **B** optimized at the SCS-MP2/QZVPP level of theory.[1]

Atom	x	y	z
S	0.159241	0.265383	−0.868816
C	0.383446	−0.106215	0.713258
Fe	−2.119054	−1.824127	1.377940
C	−2.786513	−2.140373	−0.420874
C	−1.533353	−2.793166	−0.201928
C	−1.677687	−3.669434	0.921790
C	−3.018381	−3.550337	1.403305
C	−3.705022	−2.606799	0.572336
C	−2.074840	0.097961	1.433822
C	−2.900444	−0.419733	2.481753
C	−2.121106	−1.326414	3.259291
C	−0.795486	−1.361946	2.709678

C	−0.751277	−0.462280	1.583313
H	−2.979573	−1.389868	−1.168140
H	−0.631130	−2.606912	−0.760478
H	−0.907801	−4.286617	1.353871
H	−3.430273	−4.054549	2.261168
H	−4.722599	−2.277582	0.698886
H	−2.356502	0.801496	0.668611
H	−3.946190	−0.202757	2.623525
H	−2.474638	−1.912501	4.091528
H	0.021041	−1.964106	3.064404
Fe	2.971614	−1.383697	1.756776
C	2.445829	−3.049799	2.618521
C	2.019334	−2.999320	1.251081
C	3.177175	−2.791533	0.434479
C	4.315770	−2.707264	1.296539
C	3.862981	−2.865596	2.646667
C	2.027040	−0.018248	2.745357
C	3.444228	0.146887	2.878125
C	4.005936	0.264148	1.575751
C	2.945962	0.175357	0.618365
C	1.705962	0.022137	1.334739
H	1.814685	−3.180597	3.481425
H	1.002590	−3.056092	0.903203
H	3.181920	−2.675125	−0.636188
H	5.330689	−2.519923	0.988807
H	4.478553	−2.821270	3.529270
H	1.323165	−0.112961	3.553427
H	3.992599	0.135703	3.805534
H	5.055148	0.350010	1.346705
H	3.037963	0.219628	−0.452704

[1] Total energy amounts to −3733.2164624872 hartree.

Supplementary Table S16. Cartesian coordinates (x, y, z, in Å) for isolated rotamer **C** optimized at the SCS-MP2/QZVPP level of theory.[1]

Atom	x	y	z
Fe	3.689751	5.937412	4.979562
Fe	8.018386	6.569477	1.132971
S	5.771449	8.728154	3.371262
C	5.829952	7.097514	3.166186
C	4.632834	6.261941	3.320632
C	3.275695	6.756049	3.289645
H	2.999208	7.776658	3.086971
C	2.395728	5.655078	3.543440
H	1.321543	5.711590	3.605064
C	3.183926	4.491703	3.775716
H	2.810143	3.517095	4.043017
C	4.567607	4.854865	3.654746
H	5.406411	4.199151	3.808661
C	4.817510	6.849352	6.273577
H	5.746551	7.349078	6.055422
C	4.660423	5.465547	6.603479
H	5.454481	4.744195	6.697592

C	3.261649	5.198424	6.727800
H	2.814409	4.240479	6.933095
C	2.554094	6.420150	6.480283
H	1.483673	6.538235	6.469786
C	3.517484	7.441009	6.200660
H	3.306558	8.461204	5.928797
C	7.081144	6.412733	2.819178
C	7.241089	5.137097	2.153361
H	6.448938	4.478733	1.843154
C	8.645039	4.915936	1.950789
H	9.083001	4.065074	1.455562
C	9.351936	6.042604	2.460064
H	10.418253	6.190557	2.416005
C	8.399976	6.982110	2.971927
H	8.606399	7.940557	3.416776
C	8.469935	6.309565	−0.741656
H	8.980745	5.465141	−1.172757
C	9.092823	7.482957	−0.203419
H	10.152188	7.672528	−0.162644
C	8.063965	8.332686	0.314160
H	8.206268	9.269110	0.826087
C	6.808216	7.683406	0.097299
H	5.849584	8.046401	0.428538
C	7.057891	6.436063	−0.558648
H	6.315634	5.703265	−0.826600

[1] Total energy amounts to −3733.2159792982 hartree.

Supplementary Table S17. Cartesian coordinates (x, y, z, in Å) of the atoms occupying the Fc_2CS unit cell optimized at the PBE-D/SVP level of theory.[1]

Atom	x	y	z
H	0.752446	7.811894	4.596614
C	0.874553	6.855126	4.083274
C	−0.090851	5.801724	3.999822
H	−1.091148	5.811858	4.450453
C	0.491567	4.719821	3.253062
H	0.018784	3.758627	3.027426
C	1.819711	5.099889	2.871774
H	2.512414	4.489023	2.286778
C	2.067270	6.448342	3.368286
C	3.263511	7.276738	3.201242
C	4.547799	6.662086	2.861769
C	5.686174	7.344459	2.276118
H	5.693107	8.406114	2.002430
C	4.948287	5.277668	3.072289
H	4.326990	4.487575	3.500683
C	6.313462	5.138917	2.671788
H	6.896250	4.214351	2.729748
S	3.155121	8.943940	3.415521
C	6.766312	6.406138	2.167901
H	7.761207	6.601485	1.752591
Fe	1.632415	5.144510	4.886128
C	1.071706	4.620535	6.768991

H	0.074096	4.765538	7.204990
C	2.157150	5.564912	6.815727
H	2.139447	6.552273	7.287551
C	1.522405	3.467716	6.031667
H	0.926661	2.573175	5.817735
C	2.882127	3.701585	5.623350
H	3.495839	3.014951	5.025302
C	3.273922	4.995916	6.113540
H	4.243227	5.488484	5.962171
Fe	5.144953	5.763327	1.110737
C	5.417713	4.338179	−0.318110
H	6.056578	3.456124	−0.208873
C	4.027564	4.421294	0.034788
H	3.429994	3.599169	0.446317
C	3.583760	5.760185	−0.231257
H	2.574343	6.146417	−0.048645
C	5.828252	5.627941	−0.804990
H	6.835474	5.887920	−1.147965
C	4.694781	6.512220	−0.743734
H	4.678863	7.568307	−1.039766
H	2.572266	3.509365	8.597593
C	3.045063	2.548643	8.826484
C	2.459917	1.466438	9.570465
H	1.457083	1.455106	10.015399
C	4.375852	2.170299	8.453367
H	5.071298	2.782932	7.873281
C	3.426319	0.414544	9.659879
H	3.302360	−0.542497	10.172349
C	4.622429	0.822237	8.951575
C	5.818758	−0.007118	8.787892
C	7.103835	0.606795	8.450312
C	8.242802	−0.075543	7.865336
H	8.251502	−1.137705	7.593850
C	9.321035	0.864311	7.752788
H	10.315856	0.669452	7.336599
C	8.866832	2.132266	8.253560
H	9.448172	3.057783	8.308436
C	7.502581	1.992318	8.656928
H	6.881519	2.782747	9.084926
S	5.708646	−1.674496	9.000870
Fe	4.176199	2.127526	10.466345
C	3.605778	2.655767	12.346095
H	2.607971	2.506559	12.780339
C	4.696956	1.718410	12.400465
H	4.684317	0.734039	12.878812
C	4.051679	3.807903	11.604879
H	3.450355	4.696930	11.383938
C	5.413829	3.580356	11.200805
H	6.023340	4.266725	10.598279
C	5.812010	2.290719	11.698301
H	6.784148	1.802140	11.551384
Fe	7.696624	1.500660	6.696119

C	7.963016	2.919743	5.259665
H	8.600237	3.803624	5.362881
C	6.573748	2.835241	5.615571
H	5.974181	3.657480	6.023746
C	6.132451	1.493986	5.357089
H	5.124281	1.107202	5.543673
C	8.375601	1.628493	4.778596
H	9.383063	1.368877	4.435363
C	7.244417	0.741867	4.846148
H	7.230982	−0.315640	4.555096
H	4.158096	10.875012	5.211112
C	3.561155	11.697765	5.621828
C	4.006441	13.036855	5.884450
H	5.016110	13.420897	5.700108
C	2.171084	11.616687	5.976068
H	1.531523	10.734924	5.869167
C	1.762188	12.908124	6.459992
H	0.755351	13.170179	6.802736
C	2.896399	13.791259	6.395967
H	2.913179	14.847733	6.690350
Fe	2.443869	13.038281	4.543443
C	2.641038	12.549359	2.582525
H	3.264111	11.760374	2.154725
C	1.276572	12.407500	2.984349
H	0.696944	11.480782	2.928846
C	0.820483	13.674678	3.485312
H	0.175097	13.868116	3.900412
C	3.038154	13.935039	2.790778
C	4.322734	14.550189	2.453560
C	5.519152	13.721302	2.289143
C	6.714446	14.128489	1.579103
C	5.765272	12.372692	2.786077
H	5.070814	11.760820	3.367925
H	6.838894	15.085460	1.066733
C	7.094872	11.993112	2.409922
H	7.566750	11.031844	2.637552
C	7.679800	13.075426	1.665733
H	8.681786	13.086193	1.218868
C	1.897879	14.615727	3.374967
H	1.887633	15.677885	3.647000
Fe	5.960459	12.417561	0.772351
C	4.716802	10.970621	0.034097
H	4.104112	10.283221	0.632300
C	4.322505	12.263093	−0.459034
H	3.351570	12.753467	−0.311007
C	6.078000	10.739921	−0.371032
H	6.676277	9.848042	−0.153934
C	6.527221	11.892896	−1.109070
H	7.525363	12.040409	−1.543100
C	5.439213	12.833923	−1.159876
H	5.455333	13.819950	−1.634539
S	4.431514	16.217552	2.240680

C	−0.925155	9.931316	4.485041
H	0.071965	9.784419	4.048771
C	−2.014099	8.990943	4.434569
H	−2.000247	8.005304	3.959037
C	−1.372400	11.083597	5.225211
H	−0.773257	11.974915	5.443453
C	−2.733217	10.853427	5.632142
H	−3.343622	11.539377	6.234244
C	−3.129250	9.561996	5.137610
H	−4.100049	9.071729	5.287258
Fe	−1.490095	9.404428	6.366220
C	−1.683418	9.447892	8.379729
H	−2.378483	10.058896	8.961989
C	−0.354581	9.828775	8.002026
H	0.117127	10.790481	8.228282
C	0.230438	8.747324	7.256835
H	1.231949	8.738109	6.809010
C	−0.734072	7.693375	7.170703
H	−0.610046	6.736128	6.658616
C	−1.928950	8.099255	7.882544
C	−3.124634	7.269347	8.047056
C	−4.409543	7.882452	8.386702
C	−5.547243	7.199838	8.973567
H	−5.553839	6.138527	9.248338
C	−4.810777	9.266653	8.176041
H	−4.190757	10.056814	7.746113
C	−6.175534	9.404986	8.577583
H	−6.759010	10.328861	8.518755
S	−3.014786	5.602490	7.830024
C	−6.627506	8.137938	9.082636
H	−7.621981	7.942697	9.499017
Fe	−5.005019	8.780333	10.138238
C	−5.271418	10.202985	11.570983
H	−5.907545	11.087344	11.465469
C	−3.881962	10.116138	11.216932
H	−3.281467	10.937713	10.808426
C	−3.442594	8.774958	11.478825
H	−2.434663	8.385790	11.294441
C	−5.686106	8.913472	12.054744
H	−6.694200	8.656078	12.397512
C	−4.555882	8.025425	11.990228
H	−4.543713	6.968246	12.282732

[1] Total energy amounts to −14937.0711018872 hartree.

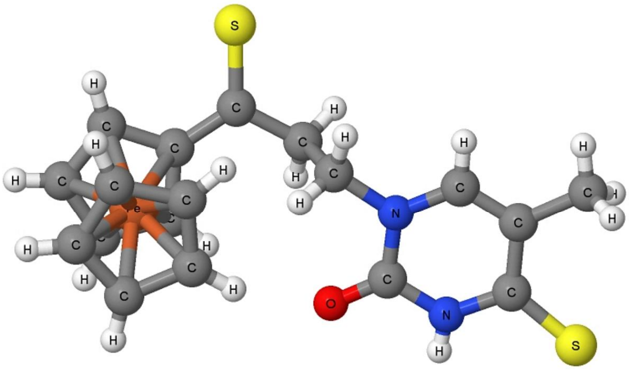

DUFYAG

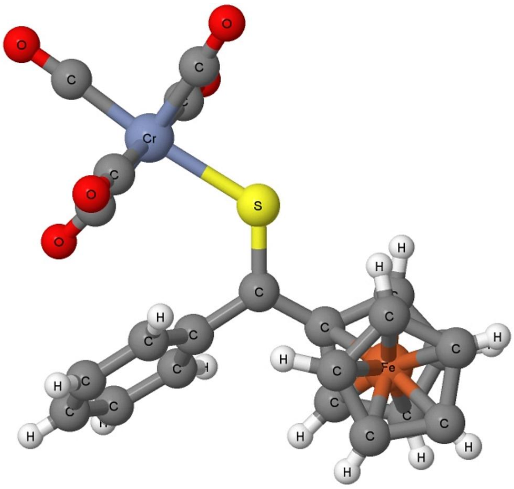

JEPVIJ

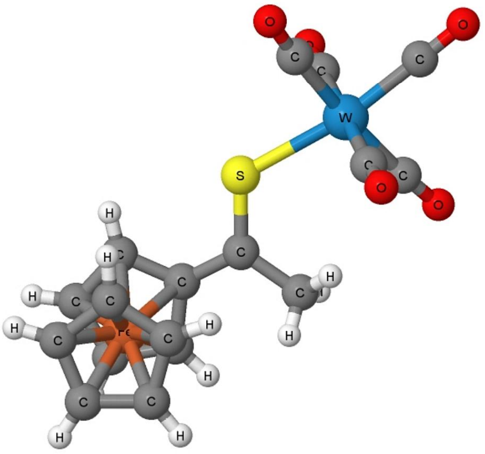

VUTKAX

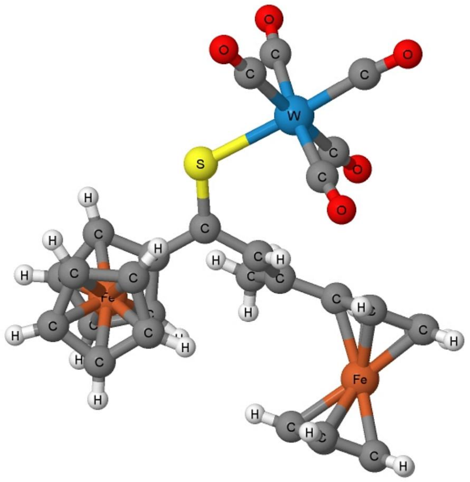

VUTKEB

Supplementary Figure S1. Molecular structures of DUFYAG ($C_{18}H_{18}Fe_1N_2O_1S_2$), JEPVIJ ($C_{22}H_{14}Cr_1Fe_1O_5S_1$), VUTKAX ($C_{17}H_{12}Fe_1O_5S_1W_1$) and VUTKEB ($C_{29}H_{22}Fe_2O_5S_1W_1$).

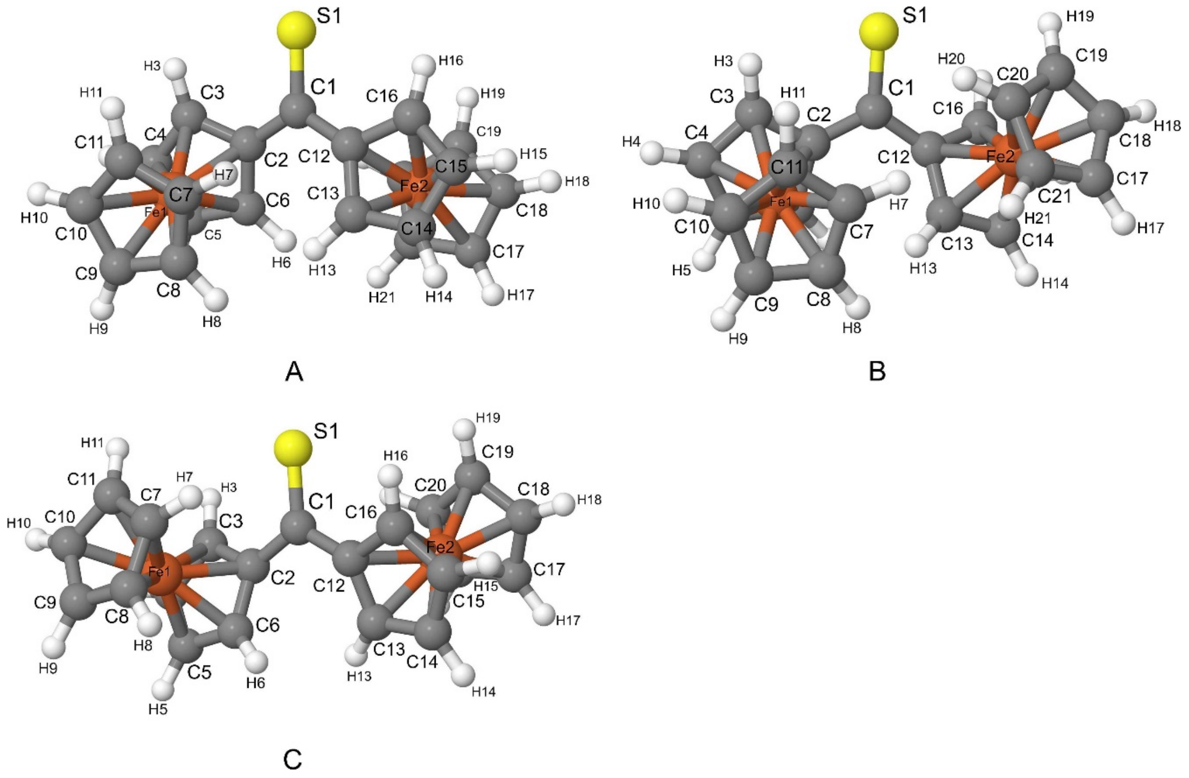

Supplementary Figure S2. Rotamers of Fc_2CS with all their atoms numbered.

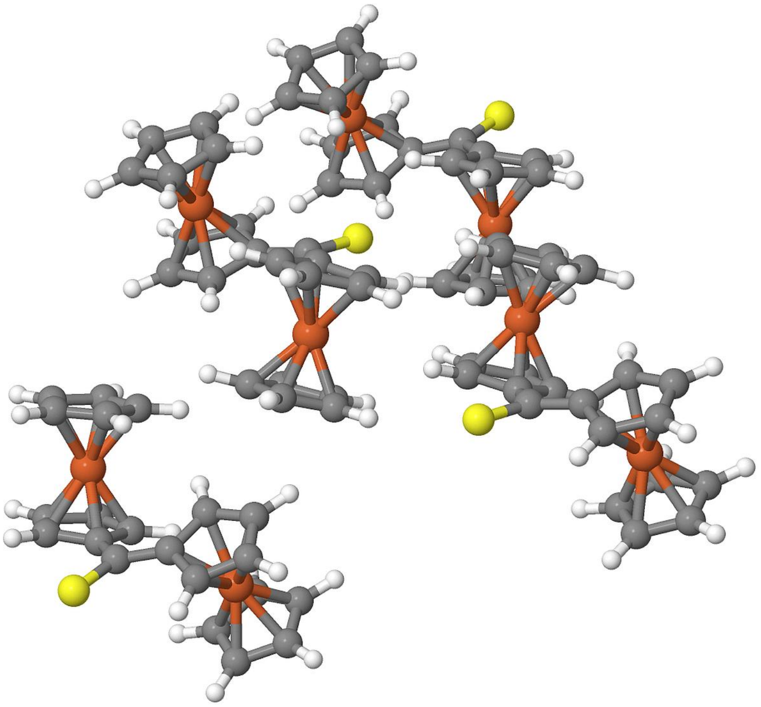

Supplementary Figure S3. Tetramer extracted from the PBE-D/SVP-optimized crystal structure of Fc_2CS.

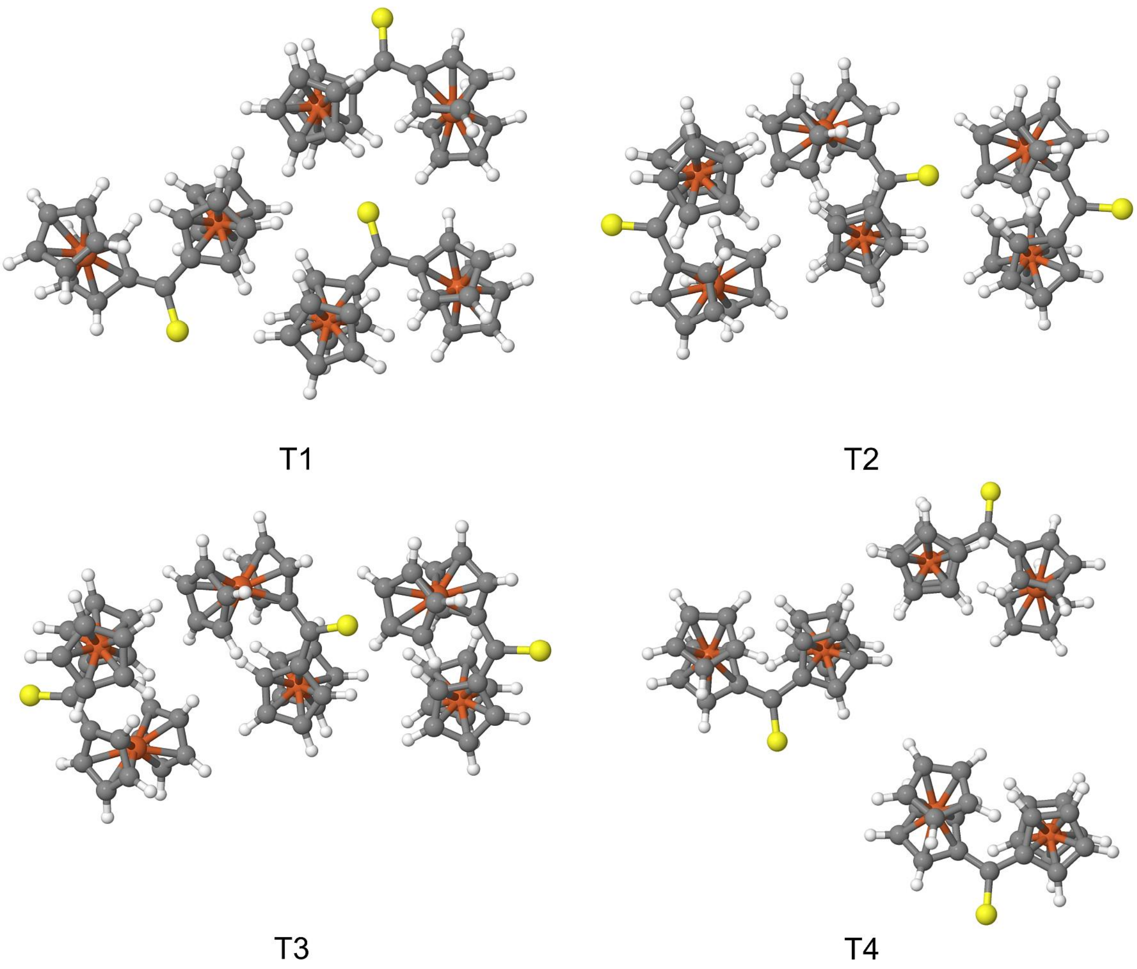

Supplementary Figure S4. Triples of molecules (that is, trimers **T1**–**T4**) extracted from the PBE-D/SVP-optimized crystal structure of Fc_2CS.

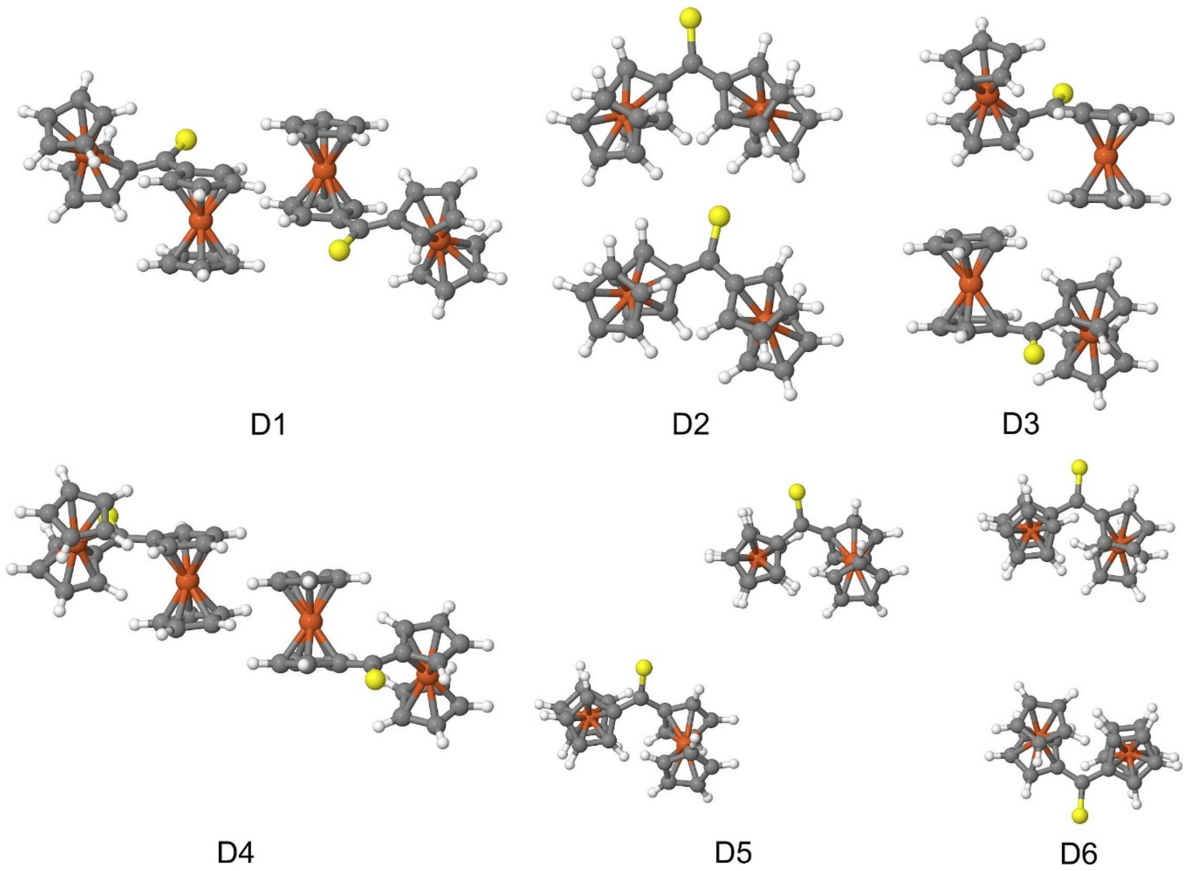

Supplementary Figure S5. Pairs of molecules (that is, dimers **D1–D6**) extracted from the PBE-D/SVP-optimized crystal structure of Fc_2CS.

References

S1. Perdew, J.P.; Schmidt, K. Jacob's ladder of density functional approximations for the exchange-correlation energy. *AIP Conf. Proc.* **2001**, *577*, 1–20.

S2. Sierka, M.; Hogekamp, A.; Ahlrichs, R. Fast evaluation of the Coulomb potential for electron densities using multipole accelerated resolution of identity approximation. *J. Chem. Phys.* **2003**, *118*, 9136– 9148.

S3. Burow, A.M.; Sierka, M.; Mohamed, F. Resolution of identity approximation for the Coulomb term in molecular and periodic systems. *J. Chem. Phys.* **2009**, *131*, 214101.

S4. Weigend, F. Accurate Coulomb-fitting basis sets for H to Rn. *Phys. Chem. Chem. Phys.* **2006**, *8*, 1057–1065.

S5. Hättig, C. Optimization of auxiliary basis sets for RI-MP2 and RI-CC2 calculation: Core-valence and quintuple-ζ basis sets for H to Ar and QZVPP basis sets for Li to Kr. *Phys. Chem. Chem. Phys.* **2005**, *7*, 59–66.

S6. Hättig, C. Geometry optimizations with the coupled-cluster model CC2 using the resolution-of-the-identity approximation. *J. Chem. Phys.* **2003**, *118*, 7751–7761.

S7. Weigend, F. A fully direct RI-HF algorithm: Implementation, optimised auxiliary basis sets, demonstration of accuracy and efficiency. *Phys. Chem. Chem. Phys.* **2002**, *4*, 4285–4291.

S8. Weigend, F.; Ahlrichs, R. Balanced basis sets of split valence, triple zeta valence and quadruple zeta valence quality for H to Rn: Design and assessment of accuracy. *Phys. Chem. Chem. Phys.* **2005**, *7*, 3297–3305.

S9. Miertus, S.; Scrocco, E.; Tomasi, J. Electrostatic interaction of a solute with a continuum. A direct utilization of ab initio molecular potentials for the prevision of solvent effects. *Chem. Phys.* **1981**, *55*, 117–129.

S10. Kabsch, W. A discussion of the solution for the best rotation to relate two sets of vectors. *Acta Cryst. A* **1978**, *34*, 827–828.

S11. Speakman, L.D.; Papas, B.N.; Woodcock, H.L.; Schaefer III, H.F. The microwave and infrared spectroscopy of benzaldehyde: Conflict between theory and experimental deductions. *J. Chem. Phys.* **2004**, *120*, 4247–4250.

S12. Godunov, I.A.; Bataev, V.A.; Abramenkov, A.V.; Pupyshev, V.I. The barriers to internal rotation of benzaldehyde and benzoyl fluoride: "Reconciliation" between theory and experiment. *J. Phys. Chem. A* **2014**, *118*, 10159–10165.

S13. Haaland, A.; Nilsson, J.E. The determination of barriers to internal rotation by means of electron diffraction. Ferrocene and ruthenocene. *Acta. Chem. Scand.* **1968**, *22*, 2653–2670.

S14. Boys, S.F.; Bernardi, F. The calculation of small molecular interactions by the differences of separate total energies. Some procedures with reduced errors. *Mol. Phys.* **1970**, *19*, 553–566.

S15. Dunning, T.H., Jr. Gaussian basis sets for use in correlated molecular calculations. I. The atoms boron through neon and hydrogen. *J. Chem. Phys.* **1989**, *90*, 1007–1023.
